# Population-Level Lambing Interval Patterns and Evaluation of Three Previously Reported Candidate SNPs in Hu and Tan Sheep

**DOI:** 10.3390/ani16162527

**Published:** 2026-08-13

**Authors:** Yuan Gou, Yi Yin, Jiaxu Hao, Qinghua Zhao, Hui Zhou, Zehu Yuan, Huiguo Yang, Kanati Shalike, Shanhe Wang, Wei Sun

**Affiliations:** 1 College of Veterinary Medicine, Yangzhou University, Yangzhou 225009, China; 2 College of Animal Science and Technology, Yangzhou University, Yangzhou 225009, China; 3 Joint International Research Laboratory of Agriculture and Agri-Product Safety, Ministry of Education of China, Yangzhou University, Yangzhou 225009, China; 4 International Joint Research Laboratory in Universities of Jiangsu Province of China for Domestic Animal Germplasm Resources and Genetic Improvement, Yangzhou University, Yangzhou 225009, China; 5 Institute of Animal Husbandry, Xinjiang Academy of Animal Sciences, Urumqi 830013, China

**Keywords:** Hu sheep, Tan sheep, lambing interval, *PAPPA*, *CD226*, *ADAMTSL1*

## Abstract

Lambing interval is an indicator of reproductive efficiency in ewes. This study characterized population-level lambing interval patterns in Hu and Tan sheep and evaluated three previously reported candidate SNPs in *ADAMTSL1*, *CD226*, and *PAPPA*. Among 256 genotyped Hu ewes and 94 genotyped Tan ewes, 244 and 92 ewes, respectively, had at least two valid lambing dates. After applying a unified quality-control range of 180–450 days, 711 intervals from 230 Hu ewes and 219 intervals from 82 Tan ewes were retained. The raw mean lambing intervals were 277.81 ± 27.56 days in Hu sheep and 368.81 ± 22.51 days in Tan sheep. After adjustment for parity, year, season, preceding litter size, and repeated ewe records, Tan sheep had an 80.99-day longer interval than Hu sheep (95% CI: 75.34–86.64 days; *p* = 3.17 × 10^−126^). This difference remained significant across all parity groups. None of the three candidate SNPs was significantly associated with adjusted lambing interval after false-discovery-rate correction. Analyses of within-ewe interval variability also yielded no supported associations, although *CD226* showed a weak, non-significant tendency toward lower variability in Hu sheep. These findings demonstrate a marked population-level difference in reproductive timing but do not support the immediate use of the tested SNPs as markers for lambing interval or within-ewe interval variability.

## Simple Summary

Lambing interval reflects the reproductive rhythm and production efficiency of ewes. This study compared population-level lambing interval patterns between Hu and Tan sheep and evaluated three candidate SNPs previously reported in ovine genomic studies. Tan sheep showed a markedly longer lambing interval than Hu sheep after adjustment for parity, year, season, preceding litter size, and repeated records within ewes. None of the three SNPs showed a robust association with lambing interval after false-discovery-rate correction. These findings demonstrate marked population-level differences in reproductive timing but do not support the use of the tested SNPs as validated markers for lambing interval.

## 1. Introduction

Reproductive efficiency is one of the most important determinants of profitability and sustainability in sheep production [1]. Among reproductive traits, lambing interval is a key indicator that reflects the time between two successive lambing events and is closely associated with postpartum recovery, rebreeding efficiency, ewe reproductive rhythm, and annual lamb production. Compared with litter size, which mainly reflects the number of lambs produced in a single lambing event, lambing interval provides additional information on the continuity and stability of ewe reproductive performance. A shorter and more stable lambing interval is generally beneficial for improving flock expansion rate and production efficiency, whereas prolonged intervals may reduce the number of lambing opportunities and increase non-productive days in breeding ewes. Previous studies have shown that lambing interval is influenced by genetic background, parity, reproductive season, nutrition, mating management, health status, and repeated variation within individual ewes [2,3].

Hu sheep and Tan sheep are two representative Chinese sheep breeds with distinct reproductive characteristics [4]. Hu sheep are well known for early maturity, year-round estrus, and relatively high reproductive performance, whereas Tan sheep are characterized by strong adaptability to harsh environments but generally exhibit a longer reproductive cycle and a lower lambing frequency [5,6]. These populations provide a useful contrast for describing reproductive timing; however, differences between them may reflect the combined effects of reproductive seasonality, mating schedules, farm management, and genetic background. Therefore, comparisons between Hu and Tan sheep should be interpreted as population-level contrasts unless breed and production environment can be fully separated. Understanding the genetic basis of lambing interval and related reproductive-timing phenotypes may help improve selection strategies beyond litter size alone, particularly in breeding systems aimed at enhancing continuous reproductive capacity [7,8].

Molecular marker-assisted selection has been widely used to improve economically important traits in livestock [9]. In sheep, several major fecundity genes, including *BMPR1B*, *BMP15*, and *GDF9*, have been extensively investigated for their effects on ovulation rate and litter size [9,10,11,12]. However, litter size and the interval between successive lambings represent biologically distinct traits. The present study therefore did not examine the classical prolificacy loci, but instead focused on SNPs previously reported in genome-wide studies of lambing interval or reproductive seasonality.

The *PAPPA* gene encodes pregnancy-associated plasma protein-A (PAPP-A), a metalloproteinase involved in the insulin-like growth factor system [13]. PAPP-A cleaves insulin-like growth factor-binding proteins, particularly IGFBP-4, thereby regulating local IGF bioavailability in ovarian follicles [14]. Studies in multiple domestic species have shown that *PAPPA* is present in preovulatory follicles and participates in IGFBP-4 proteolytic degradation, while functional studies in mice have suggested that loss of *PAPPA* compromises ovarian steroidogenesis and female fertility [14]. More importantly for the present study, a SNP within *PAPPA* was previously associated with lambing interval in a whole-genome sequencing study of 53 Tibetan sheep and was subsequently evaluated in 1130 animals, providing a direct rationale for its independent assessment [15].

*CD226* is an immune regulatory molecule involved in immune cell activation and cell–cell interaction. Successful reproduction requires a finely regulated immune balance during postpartum recovery, uterine repair, and pregnancy establishment [16]. Recent studies have highlighted the importance of immune checkpoint molecules, including *CD226*-related pathways, in immune regulation during pregnancy [17]. The *CD226* locus was previously implicated in reproductive seasonality in a Genome-wide association study (GWAS) of 205 Rasa Aragonesa ewes [18]. The rs404360094 variant is a nonsynonymous exon variant causing an asparagine-to-aspartic-acid substitution. Although these findings support its evaluation as a candidate marker, they do not establish a direct effect on lambing interval in other sheep populations.

*ADAMTSL1* belongs to the ADAMTS-like protein family and is functionally related to extracellular matrix organization and tissue remodeling [19]. Members of the ADAMTS family have been implicated in ovarian follicle remodeling, cumulus–oocyte complex expansion, ovulation, implantation, and reproductive tissue dynamics [20,21]. For example, *ADAMTSL1* lacks protease activity but may influence extracellular-matrix organization through interactions with fibrillin-containing microfibrils. Its biological connection with reproductive tissue remodeling is therefore plausible but indirect. More importantly, rs415581052, previously designated s50067.1, was significantly associated with lambing interval in a GWAS of 122 Lori-Bakhtiari ewes [22]. These findings provide both biological and genetic support for evaluating this SNP in additional sheep populations.

Therefore, the present study aimed to characterize population-level lambing interval patterns in the studied Hu and Tan sheep populations and to independently evaluate three previously reported candidate SNPs. Specifically, we examined population and parity-related patterns using repeated-record mixed models, described genotype and allele frequencies, and tested population-specific associations with lambing interval and within-ewe interval variability.

## 2. Materials and Methods

### 2.1. Animals and Reproductive Records

Hu sheep and Tan sheep populations with individual reproductive records and candidate SNP genotype information were used in this study. Hu sheep were obtained from the Suzhou Breeding Sheep Farm in Jiangsu Province, China, and their original reproductive records covered the period from 2017 to 2025. Tan sheep were obtained from a Tan sheep farm in the Ningxia Hui Autonomous Region, China, and their reproductive records covered the period from 2019 to 2024. Both flocks were maintained primarily for meat production under planned breeding programs. Breeding groups were generally established by assigning one selected breeding ram to approximately 15–25 ewes, and pedigree information for the resulting offspring was routinely recorded. Ewes were generally retained for breeding until approximately 6–7 years of age, subject to reproductive performance, health status, and body condition. Culling criteria included failure to conceive for two consecutive years, litter size consistently below the breed-specific average, a body condition score persistently below 2 on a five-point scale, incurable chronic disease, poor mammary development, or mastitis severe enough to prevent adequate nursing of lambs. Detailed historical information on the exact timing and duration of ram introduction was not consistently available across all recording years for both flocks. Therefore, mating-management effects could not be fully separated from population and farm effects. The reproductive records included ewe identification, lambing dates, parity information, and continuous lambing history. Lambing records were organized chronologically for each ewe before calculating lambing intervals. The lambing interval was defined as the number of days between two consecutive lambing events of the same ewe. Records with missing ewe identification, missing lambing dates, duplicated entries, inconsistent parity order, or biologically unreasonable values were checked and excluded before downstream analysis. All available lambing dates were sorted chronologically within each ewe, and lambing interval was defined as the number of days between two adjacent recorded lambing events, irrespective of calendar year. Therefore, two consecutive lambing events occurring within the same calendar year were retained when they met the predefined quality-control criteria. The lower threshold of 180 days was used to exclude biologically implausible intervals, duplicated records, or potential date-entry errors and did not represent a postpartum conception cutoff or a definition of twice-yearly lambing. The primary analysis used a prespecified interval range of 180–450 days. To evaluate whether the findings depended on the upper threshold, sensitivity analyses were additionally performed using expanded ranges of 180–730 and 180–900 days. Because detailed information on ram exposure, mating dates, pregnancy diagnosis, conception failure, abortion, pregnancy loss, and missed lambing events was not consistently available, prolonged intervals could not be separated into delayed breeding opportunity, reproductive failure, pregnancy loss, skipped breeding seasons, or incomplete recording. All experimental procedures involving sheep were approved by the Experimental Animal Management Committee of Yangzhou University (Approval No. NSFC2020-NFY-1).

### 2.2. Calculation of Lambing Interval and Within-Ewe Variability Metrics

For each ewe, the lambing interval was calculated using the following formula:

Lambing interval = date of subsequent lambing − date of previous lambing

The interval between adjacent parities was recorded according to the order of lambing events, such as 1–2, 2–3, 3–4 and subsequent parity intervals. Individual adjacent-parity intervals were used as the primary observational units, and repeated intervals from the same ewe were accounted for using a random ewe effect. The definitions of the ewe-level summary and within-ewe variability metrics are provided in Table 1.

To characterize variation in repeated lambing intervals within individual ewes, three within-ewe variability metrics were calculated: interval standard deviation (SD), scaled median absolute deviation (MAD), and coefficient of variation (CV). These metrics were calculated only for ewes with at least three valid lambing intervals. Interval SD and MAD describe the absolute dispersion of repeated intervals, whereas CV describes dispersion relative to the ewe-specific mean interval and was calculated as follows:

$$IntervalCV=\frac{IntervalSD}{Meaninterval}$$

Scaled MAD was calculated as:

$$MAD=1.4826\times median\left(\left|x_{i}-median(x)\right|\right)$$

These measures were treated as descriptive and exploratory indicators of within-ewe lambing-interval variability rather than as validated biological stability traits or predictors of future reproductive success. Because CV and the other variability measures may depend on the ewe-specific mean interval and the number of available records, subsequent association models were adjusted for each ewe’s mean lambing interval and number of valid intervals.

### 2.3. Candidate SNP Selection

The three candidate SNPs were selected from previously published ovine genome-wide studies. The *ADAMTSL1* rs415581052 locus was reported in a lambing-interval GWAS of 122 Lori-Bakhtiari ewes [22], *CD226* rs404360094 was evaluated following a reproductive-seasonality GWAS of 205 Rasa Aragonesa ewes [18], and the *PAPPA* rs420485231 locus was identified in a whole-genome sequencing study of 53 Tibetan sheep and subsequently evaluated in 1130 animals [15]. The SNPs examined in the present study corresponded to the variants reported in those studies. The genomic positions, alleles, annotations, and validation primers for the three candidate SNPs are summarized in Table 2.

### 2.4. Genomic DNA Extraction and Quality Assessment

Whole-blood samples were collected from the jugular vein of each ewe into sterile vacuum tubes containing EDTA anticoagulant, with approximately 2 mL collected from each animal. Immediately after collection, the tubes were gently inverted several times to ensure adequate mixing with the anticoagulant. The samples were transported to the laboratory at approximately 4 °C within 3 h of collection and were immediately stored at −80 °C upon arrival. Before DNA extraction, each frozen blood sample was removed from −80 °C storage and thawed once at room temperature until completely liquefied. The sample was then immediately placed on ice, gently inverted to obtain a homogeneous suspension, and processed without further freeze–thaw cycles. Genomic DNA was extracted from whole blood using the phenol–chloroform method. Unless otherwise specified, all centrifugation steps were performed at 4 °C and 12,000 rpm for 10 min using a refrigerated high-speed centrifuge (Eppendorf SE, Hamburg, Germany). Briefly, 1–2 mL of thawed whole blood was transferred into a sterile 2 mL centrifuge tube and centrifuged. After removal of the supernatant, the cell pellet was washed with 1.5 mL of PBS, thoroughly resuspended using a Vortex-Genie 2 Mixer (Scientific Industries, Inc., Bohemia, NY, USA), gently mixed on ice for 20 min, and centrifuged again. The PBS-washing procedure was repeated once. After removal of the supernatant, the pellet was thoroughly dispersed using a sterile pipette tip. Subsequently, 500 μL of laboratory-prepared DNA lysis buffer containing 10 mM Tris, 100 mM disodium EDTA dihydrate, and 0.5% sodium dodecyl sulfate (SDS), adjusted to pH 8.0, and 6 μL of proteinase K solution (Beijing Solarbio Science & Technology Co., Ltd., Beijing, China) were added to each tube. The lysis buffer had been sterilized by autoclaving, stored at 4 °C, and prewarmed at 37 °C until completely dissolved before use. Samples were incubated overnight at 37 °C with gentle shaking using a THZ-C thermostatic shaker (Suzhou Peiying Experimental Equipment Co., Ltd., Suzhou, China) until the pellets were completely lysed and the solutions became clear. After complete lysis, 1 mL of DNA-saturated phenol (Beijing Solarbio Science & Technology Co., Ltd., Beijing, China) was added to each sample. The mixture was gently inverted on ice for 20 min and centrifuged. The upper aqueous phase was carefully transferred into a new sterile 2 mL centrifuge tube and mixed with 500 μL of DNA-saturated phenol and 500 μL of chloroform. After gentle mixing on ice for 20 min and centrifugation, the upper aqueous phase was transferred into a new tube and extracted once more with 1 mL of chloroform under the same conditions. The final aqueous phase was transferred into a sterile 1.5 mL centrifuge tube and mixed with 1 mL of prechilled absolute ethanol. The tubes were gently inverted until visible flocculent DNA precipitates formed and were then incubated at −20 °C for 20–30 min. After centrifugation at 4 °C and 12,000 rpm for 10 min, the supernatant was discarded. The DNA pellet was washed twice with 1 mL of prechilled 75% ethanol. For each wash, the pellet was gently mixed on ice for 20 min and centrifuged at 4 °C and 12,000 rpm for 10 min. After the final wash, residual ethanol was removed, and the DNA pellet was air-dried at room temperature for approximately 30 min and dissolved in 50 μL of nuclease-free water. DNA concentration and purity were measured using a NanoReady microvolume UV–Vis spectrophotometer (Hangzhou LifeReal Biotechnology Co., Ltd., Hangzhou, China). DNA integrity was evaluated by 1% agarose gel electrophoresis. Samples with no obvious degradation, an OD260/OD280 ratio of 1.8–2.0, and a DNA concentration greater than 20 ng/μL were considered suitable for subsequent PCR amplification, Sanger sequencing validation, and PARMS genotyping. Qualified DNA samples were stored at −80 °C until further analysis.

### 2.5. PCR Amplification and Sanger Sequencing Procedures

For each candidate SNP, primers were designed based on the flanking genomic sequence of the target locus. PCR amplification was performed using genomic DNA extracted from Hu and Tan sheep blood samples as the template. PCR amplification was performed using a T100™ Thermal Cycler (Bio-Rad Laboratories, Inc., Hercules, CA, USA). PCR amplification was performed in a total reaction volume of 50 μL containing 1 μL of genomic DNA, 1 μL of each primer (10 μM), 25 μL of 2× PCR Master Mix (Tsingke Biotechnology Co., Ltd., Nanjing, China), and 22 μL of nuclease-free water. The amplification program consisted of an initial denaturation at 94 °C for 15 min; 10 touchdown cycles of denaturation at 94 °C for 20 s and annealing/extension for 1 min, with the temperature beginning at 65 °C and decreasing by 0.8 °C per cycle to approximately 57 °C; followed by 32 cycles of denaturation at 94 °C for 20 s and annealing/extension at 57 °C for 1 min. PCR products were examined by electrophoresis on 1% agarose gels stained with a nucleic acid dye. Products showing clear bands of the expected size were submitted to Tsingke Biotechnology Co., Ltd. (Nanjing, China) for Sanger sequencing. Sequencing chromatograms were inspected using SnapGene version 6.0.2 (GSL Biotech LLC, Chicago, IL, USA), and genotype calls at the target nucleotide positions were manually verified. The primer sequences used for PCR amplification and Sanger sequencing validation are listed in Table 2. For each SNP, representative samples from all three genotype classes were validated by Sanger sequencing.

### 2.6. Fluorescence-Based PARMS SNP Genotyping

Genotyping of the three candidate SNPs was outsourced to Wuhan Jingtai Biotechnology Co., Ltd. (Wuhan, China) and performed using the penta-primer amplification refractory mutation system (PARMS), with genomic DNA extracted from sheep blood samples used as the template. Each reaction was conducted in a total volume of 10 μL containing 5 μL of 2× PARMS Master Mix, 0.15 μL of Allele X-specific primer (10 μM; final concentration, 150 nM), 0.15 μL of Allele Y-specific primer (10 μM; final concentration, 150 nM), 0.40 μL of the common primer (10 μM; final concentration, 400 nM), 10–100 ng of genomic DNA, and nuclease-free water to the final volume. Amplification was performed using an ABI GeneAmp PCR System 9700 (Applied Biosystems, Foster City, CA, USA) in 384-well format. The thermal cycling program consisted of initial denaturation at 94 °C for 15 min, followed by 10 touchdown cycles of denaturation at 94 °C for 20 s and annealing/extension for 1 min, with the temperature decreasing from 65 °C to 57 °C by 0.8 °C per cycle. This was followed by 32 cycles of denaturation at 94 °C for 20 s and annealing/extension at 57 °C for 1 min. After amplification, endpoint fluorescence signals were measured using a Tecan Infinite M1000 microplate reader (Tecan Group Ltd., Männedorf, Switzerland). Fluorescence signals were processed using the SNPdecoder online tool, and genotypes were assigned according to the resulting fluorescence-clustering patterns. The PARMS primer sequences used for genotyping the three candidate SNPs are provided in Table 3.

### 2.7. Genotype and Allele Frequency Analysis

For each candidate SNP, genotype frequency and allele frequency were calculated separately within Hu sheep and Tan sheep. Genotype frequency was calculated as follows:

$$f_{AA}=\frac{N_{AA}}{N}$$

For a biallelic SNP with genotypes AA, AG and GG, allele frequencies were calculated as follows:

$$p_{A}=\frac{2N_{AA}+N_{AG}}{2N}$$

$$p_{G}=\frac{2N_{GG}+N_{AG}}{2N}$$

where NAA, NAG and NGG represent the numbers of the three genotypes, and N represents the total number of genotyped individuals. Detailed genotype counts and allele frequencies are provided in Table S1.

### 2.8. Genetic Polymorphism and Hardy–Weinberg Equilibrium Analysis

Genetic diversity parameters were calculated for each SNP in each population, including observed heterozygosity (Ho), expected heterozygosity (He), effective number of alleles (Ne) and polymorphism information content (PIC). Observed heterozygosity was calculated as the proportion of heterozygous individuals in the population:

$$H_{o}=\frac{N_{het}}{N}$$

where $N_{het}$ is the number of heterozygous individuals and N is the total number of successfully genotyped individuals. Expected heterozygosity was calculated as:

$$H_{e}=1-\sum_{i=1}^{k}p_{i}^{2}$$

The effective number of alleles was calculated as:

$$N_{e}=\frac{1}{\sum_{i=1}^{k}p_{i}^{2}}$$

Polymorphism information content was calculated as:

$$PIC=1-\sum_{i=1}^{k}p_{i}^{2}-2\sum_{i=1}^{k-1}\sum_{j=i+1}^{k}p_{i}^{2}p_{j}^{2}$$

where pi and pj represent the frequencies of the i-th and j-th alleles, respectively, and k is the number of alleles at the locus. For the biallelic SNPs examined in this study, PIC can equivalently be expressed as:

$$PIC=2p_{A}p_{G}\left(1-p_{A}p_{G}\right)$$

Hardy–Weinberg equilibrium was evaluated separately for each SNP within each population using an exact test implemented in the HardyWeinberg package in R. A nominal *p* value below 0.05 was considered evidence of deviation from Hardy–Weinberg equilibrium.

### 2.9. Statistical Analysis

The lambing interval was first analyzed using a linear mixed-effects model to estimate the overall population difference. Population, parity group, year of the preceding lambing, season of the preceding lambing, and preceding litter size were included as fixed effects, whereas ewe identity was fitted as a random intercept to account for repeated records. A separate parity-specific mixed-effects model additionally included the population-by-parity interaction. Population had two levels (Hu and Tan), parity group had four levels (1–2, 2–3, 3–4, and 4+), year of the preceding lambing had eight levels in the analyzed dataset, and season had four levels. Preceding litter size was fitted as a continuous covariate. Mixed-effects models were fitted using maximum likelihood estimation (REML = FALSE). Denominator degrees of freedom and *p* values for fixed effects were calculated using the Satterthwaite approximation. Estimated marginal means and corresponding 95% confidence intervals were obtained for the Hu and Tan sheep populations. Population contrasts were estimated within each parity group, and the four parity-specific contrast tests were adjusted using the Benjamini–Hochberg false discovery rate procedure. Several sensitivity analyses were conducted to evaluate the robustness of the population-level comparison. First, a winter-matched analysis was restricted to records from the overlapping recording years 2019–2023 for which the preceding lambing occurred between December and February. Second, the primary quality-control range of 180–450 days was expanded to 180–730 days and 180–900 days to assess whether the estimated population difference depended on the original upper threshold. Third, the population-by-parity comparison was additionally evaluated using a Gaussian generalized estimating equation model with an identity link and a first-order autoregressive [AR(1)] working correlation structure, fitted using the geepack:geeglm function. Ewe identity was specified as the clustering variable, and the chronological order of lambing intervals within each ewe was used as the within-ewe time index. Robust sandwich standard errors were used. The parity-specific population contrasts obtained from this model were compared with those from the primary random-intercept mixed model. Seasons were defined according to the date of the preceding lambing as winter (December–February), spring (March–May), summer (June–August), and autumn (September–November). A formal population-by-season interaction was not fitted: the Tan records comprised almost exclusively winter lambings, with only two spring intervals and no summer or autumn intervals, so the resulting empty and sparse cells would not support a stable or interpretable interaction term. The winter-matched analysis described above was therefore used as the seasonal sensitivity analysis in its place. Candidate-SNP associations with lambing interval were initially analyzed separately within the Hu and Tan sheep populations. For each SNP, the number of G alleles was coded as 0, 1, or 2 and fitted as an additive genetic effect. The population-specific mixed models included parity group, year of the preceding lambing, season of the preceding lambing, and preceding litter size as fixed effects, with ewe identity fitted as a random intercept. To formally evaluate whether SNP effects differed between populations, an additional combined-population mixed model was fitted for each locus, including population, SNP dosage, and the population-by-SNP interaction, together with parity group, year of the preceding lambing, season of the preceding lambing, and preceding litter size as fixed effects and ewe identity as a random intercept. Prespecified sensitivity analyses combining rare genotype classes were also conducted when individual genotype groups contained few animals. Analyses of within-ewe lambing-interval variability were restricted to ewes with at least three valid intervals. Interval standard deviation (SD), scaled median absolute deviation (MAD), and coefficient of variation (CV) were transformed using

log(1+x)=ln(x+1)

To accommodate zero values and reduce right skewness. The transformed variability metrics were analyzed using linear models adjusted for each ewe’s mean lambing interval and number of valid intervals. Population-specific additive SNP associations were evaluated for each metric. These analyses were considered exploratory assessments of within-ewe variability rather than analyses of independently validated biological stability traits. For the Hu sheep *CD226* combined-genotype sensitivity analysis, animals carrying the minor allele were compared with the common homozygous group. Genotype labels were permuted 10,000 times to evaluate the robustness of the estimated differences in interval SD, MAD, and CV. The six primary population-specific SNP association tests for lambing interval, representing three SNPs in two populations, were treated as one multiple-testing family. The 18 population-specific additive tests of within-ewe variability, representing three SNPs, three variability metrics, and two populations, were treated as another family. The three *CD226* combined-genotype sensitivity tests for interval SD, MAD, and CV were treated as a separate multiple-testing family. The three population-by-SNP interaction tests for lambing interval were treated as a further family, as were the four parity-specific population contrasts obtained from the AR(1) sensitivity model. Within each family, *p* values were adjusted using the Benjamini–Hochberg false discovery rate procedure. Statistical significance was defined as an FDR-adjusted *p* < 0.05. Model assumptions were evaluated using residual-versus-fitted plots and normal Q–Q plots. Model convergence and singularity were also examined for mixed-effects models. Detailed results of the combined-category sensitivity analyses for rare genotype classes are provided in Table S2. Results of the exploratory analyses of within-ewe lambing-interval variability, including the CD226 combined-genotype and permutation analyses, are provided in Table S3. Sensitivity analyses using the expanded lambing-interval ranges are summarized in Table S4. Population-by-SNP interaction analyses and the AR(1) repeated-measures sensitivity analysis are provided in Table S5. Analyses were performed using R version 4.5.2 with the lme4, lmerTest, emmeans, geepack, HardyWeinberg, ggplot2, and ragg packages [23,24,25,26,27,28]. Figures were exported as 1000-dpi TIFF files. The R scripts used for the statistical analyses and figure generation are provided in File S1.

## 3. Results

### 3.1. Quality Control and Overall Distribution of Lambing Interval

**Figure 1:**
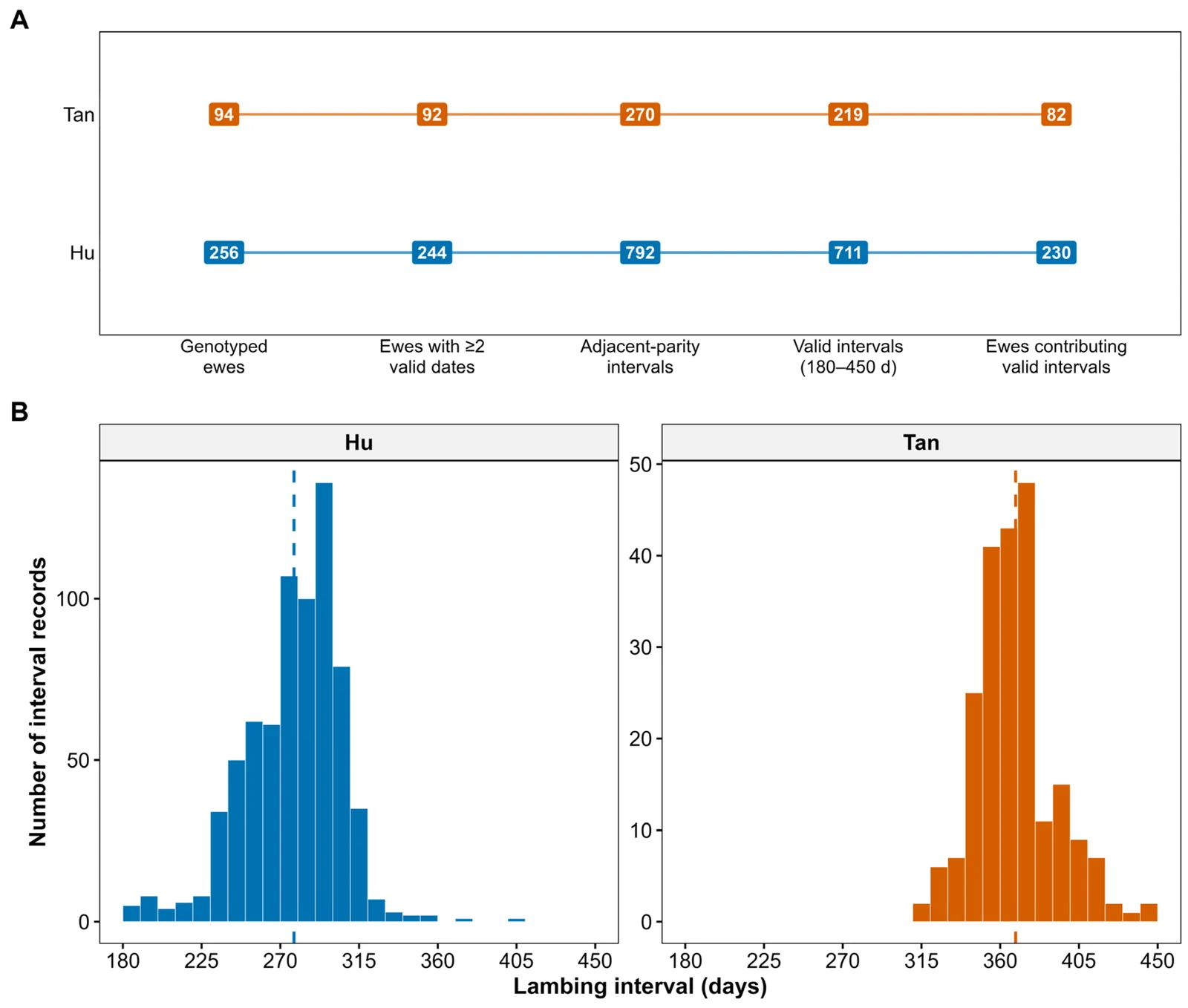
Data-processing procedure and distributions of valid lambing intervals in Hu and Tan sheep. (**A**) Data-processing and quality-control flow for the Hu and Tan sheep populations. The numbers indicate the counts of genotyped ewes, ewes with at least two valid lambing dates, adjacent-parity records, valid lambing intervals retained after applying the unified range of 180–450 days, and ewes contributing at least one valid interval. (**B**) Frequency distributions of valid lambing intervals in Hu and Tan sheep. Histograms were constructed using 10-day bins, and the vertical dashed lines indicate the unadjusted population means.

As shown in Figure 1A, genotype data were available for 256 Hu ewes and 94 Tan ewes. Of these, 244 Hu ewes and 92 Tan ewes had at least two valid lambing dates, yielding 792 and 270 adjacent-parity records, respectively. Following the application of a unified quality-control range of 180–450 days, 711 valid lambing intervals from 230 Hu ewes and 219 valid intervals from 82 Tan ewes were retained for subsequent analyses. The distributions of lambing intervals differed markedly between the two populations (Figure 1B). Hu sheep had a mean lambing interval of 277.81 ± 27.56 days, a median of 282 days, an interquartile range of 35 days, and an overall range of 180–405 days. In comparison, Tan sheep had a mean interval of 368.81 ± 22.51 days, a median of 366 days, an interquartile range of 23 days, and a range of 315–444 days. Lambing intervals in Hu sheep were distributed more broadly and were concentrated mainly between approximately 250 and 310 days, whereas those in Tan sheep were more narrowly distributed and concentrated predominantly between approximately 340 and 390 days. Overall, the unadjusted distributions indicated substantially longer lambing intervals in the Tan population than in the Hu population. Formal evaluation of this population difference was subsequently performed using mixed models accounting for temporal, reproductive, and repeated-measure effects. Among the original adjacent records, 27 Hu-sheep intervals were shorter than 180 days, whereas no Tan-sheep interval was below this threshold. In the primary analysis, 164 intervals from 148 Hu ewes and 44 intervals from 40 Tan ewes occurred within the same calendar year and were retained. In the 180–730-day sensitivity analysis, 759 intervals from 239 Hu ewes and 240 intervals from 87 Tan ewes were retained, and the adjusted Tan–Hu difference was 80.95 days (95% CI: 61.66–100.24; *p* < 0.001). In the broader 180–900-day analysis, the adjusted difference was 106.27 days (95% CI: 82.17–130.36; *p* < 0.001). Thus, the direction and statistical significance of the population contrast were robust, although inclusion of longer intervals increased the estimated magnitude of the difference. Detailed population-contrast results for the primary and expanded lambing-interval ranges are provided in Table S4.

### 3.2. Population Differences in Lambing Interval

**Figure 2:**
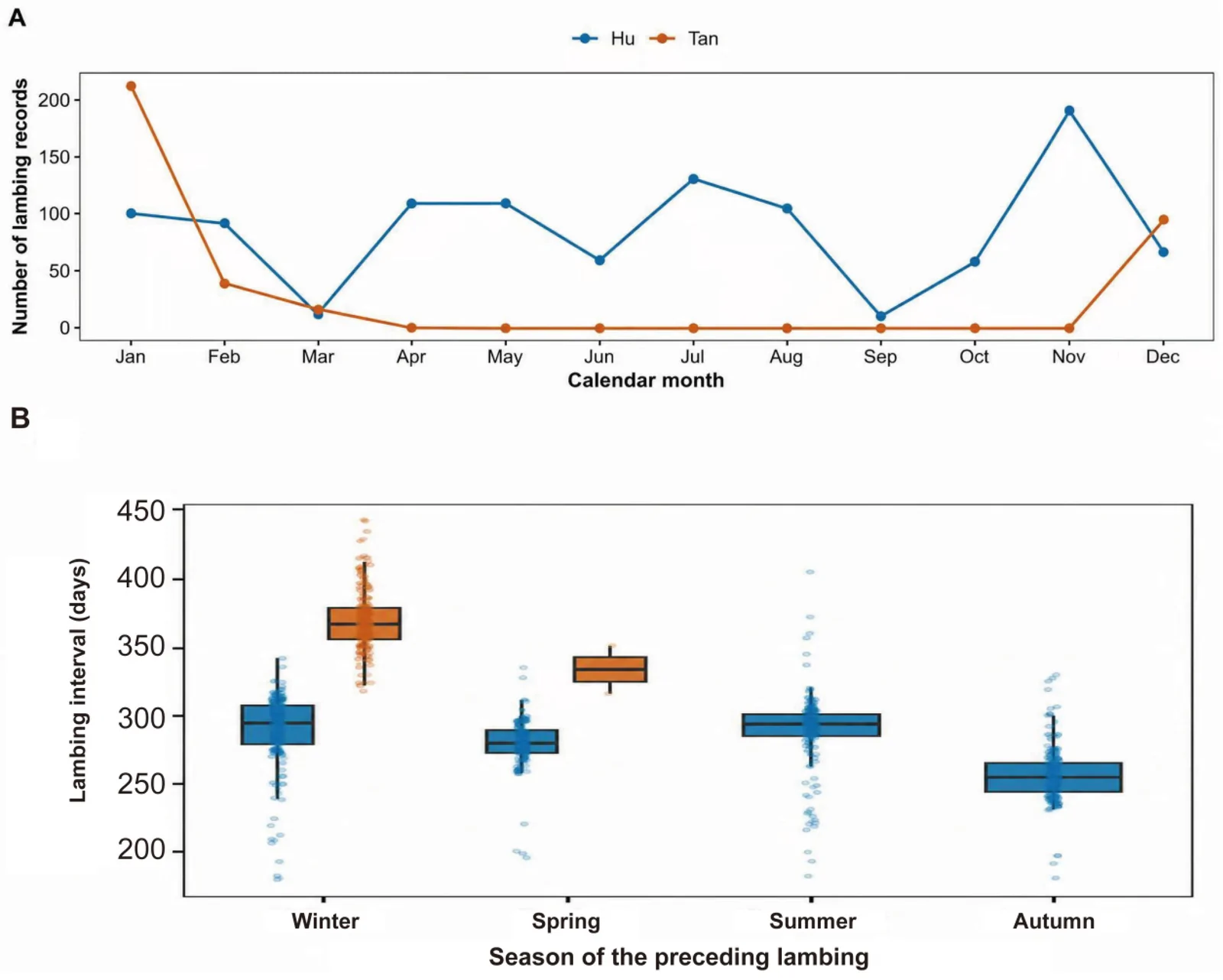
Monthly and seasonal distributions of lambing records and lambing intervals in Hu and Tan sheep. (**A**) Monthly distribution of lambing records in the two populations. (**B**) Distribution of valid lambing intervals according to the season of the preceding lambing. Box plots show the median and interquartile range; individual points represent single lambing-interval records. Seasons were defined as winter (December–February), spring (March–May), summer (June–August), and autumn (September–November).

Monthly lambing records and interval distributions according to the season of the preceding lambing are shown in Figure 2A,B. After adjustment for parity, year, season, preceding litter size, and repeated ewe records, Tan sheep had an 80.99-day longer lambing interval than Hu sheep (95% CI: 75.34–86.64 days; *p* = 3.17 × 10^−126^; Table 4). A comparable difference was observed in the winter-matched analysis of records from 2019 to 2023 (81.65 days; 95% CI: 73.37–89.93 days; *p* = 1.88 × 10^−56^). The population difference remained pronounced after restriction to winter records from the overlapping recording years, indicating that it was not explained by differences in recording periods or by the predominance of winter lambings in the Tan sheep population.

### 3.3. Parity-Specific Patterns of Lambing Interval

**Figure 3:**
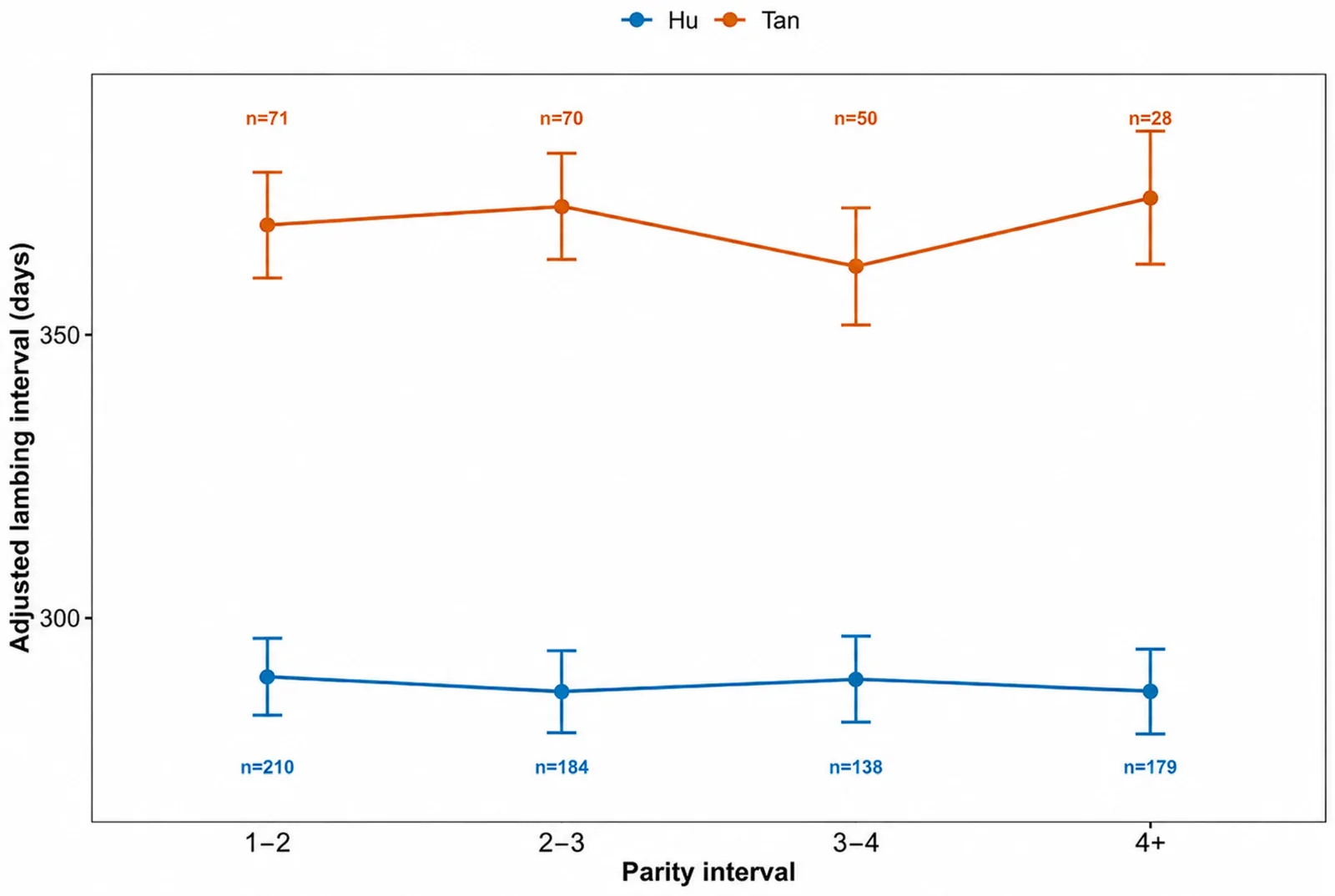
Adjusted lambing intervals across parity groups in Hu and Tan sheep. Points represent estimated marginal means, and error bars indicate 95% confidence intervals. Values labelled above or below the points indicate the number of valid lambing interval records included in each parity group. Adjusted means were estimated using a linear mixed model including population, parity group, year, season of the preceding lambing, preceding litter size, and the population-by-parity interaction as fixed effects, with ewe identity fitted as a random intercept.

Adjusted lambing intervals remained consistently longer in Tan sheep than in Hu sheep across all parity groups (Figure 3; Table 5). The Tan–Hu differences ranged from 72.73 to 86.93 days and remained significant after FDR correction. Population, year, season of the preceding lambing, and the population-by-parity interaction significantly affected lambing interval, whereas parity group and preceding litter size showed no significant main effects (Table 6). The parity-specific Tan–Hu contrasts remained similar under the AR(1) sensitivity model, ranging from 74.11 to 87.55 days, and all remained statistically significant after FDR correction (Table S5).

### 3.4. PCR Amplification and Sanger Sequencing Validation of Candidate SNPs

**Figure 4:**
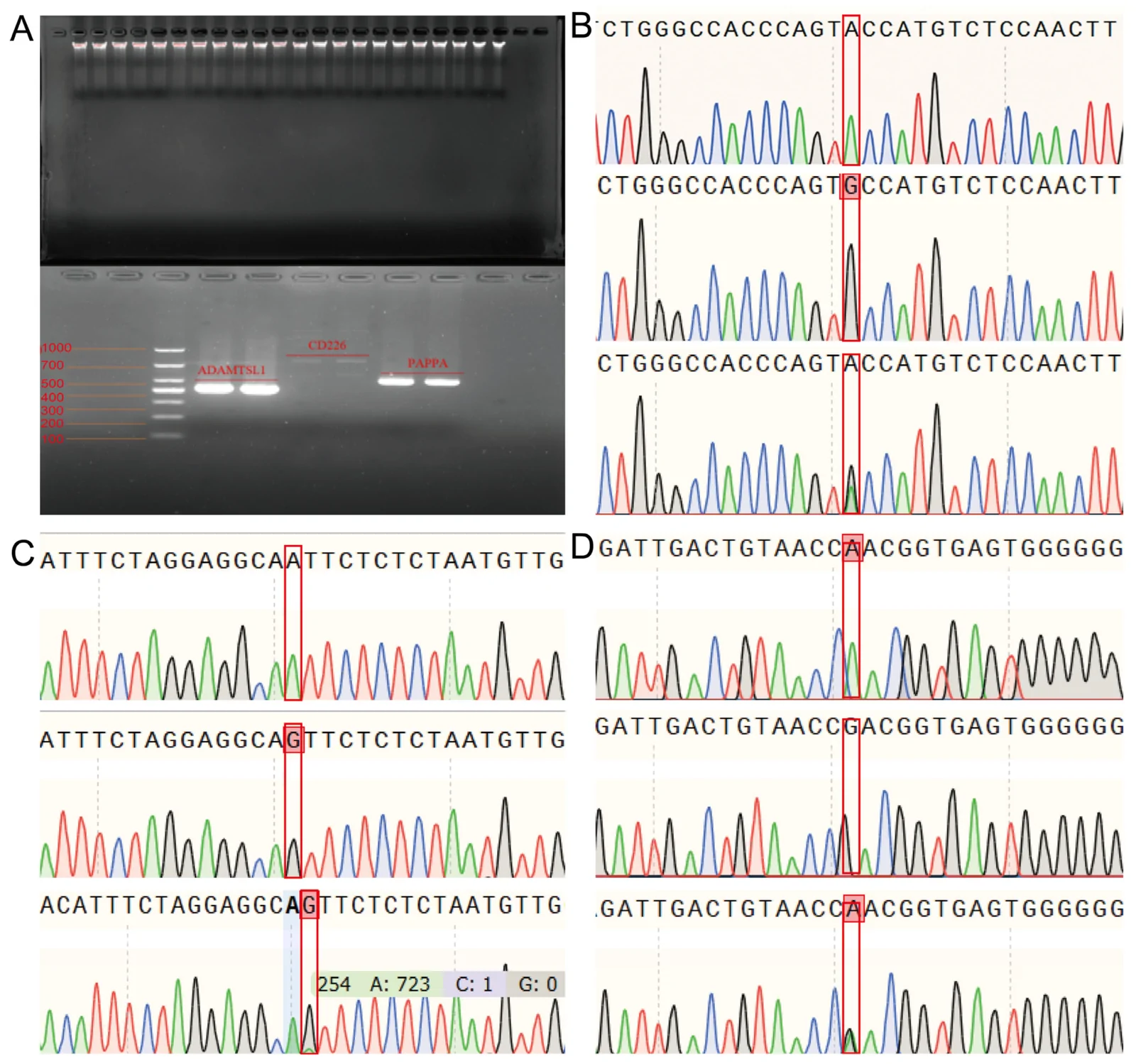
PCR amplification and Sanger sequencing validation of the three candidate SNPs. (**A**) Agarose gel electrophoresis of genomic DNA and representative PCR products for the *ADAMTSL1*, *CD226*, and *PAPPA* loci. M indicates the DNA molecular-weight marker. (**B**–**D**) Representative Sanger sequencing chromatograms for the three genotype classes at the candidate SNPs in ADAMTSL1, PAPPA, and CD226, respectively. The polymorphic nucleotide positions are highlighted by red boxes. Homozygous genotypes show a single nucleotide peak, whereas heterozygous genotypes show overlapping double peaks at the variant site. In the sequencing chromatograms, A, T, C, and G are shown in green, red, blue, and black, respectively.

Agarose gel electrophoresis showed clear amplification products of the expected sizes for the *ADAMTSL1*, *CD226*, and *PAPPA* loci, with no obvious nonspecific bands (Figure 4A). Sanger sequencing further confirmed the three genotype classes at each locus (Figure 4B–D). Homozygous individuals exhibited single nucleotide peaks at the target positions, whereas heterozygous individuals showed overlapping double peaks. The sequencing results were consistent with the corresponding genotype assignments, supporting the reliability of subsequent PARMS genotyping and association analyses.

### 3.5. Genotype and Allele Frequency Distribution

**Figure 5:**
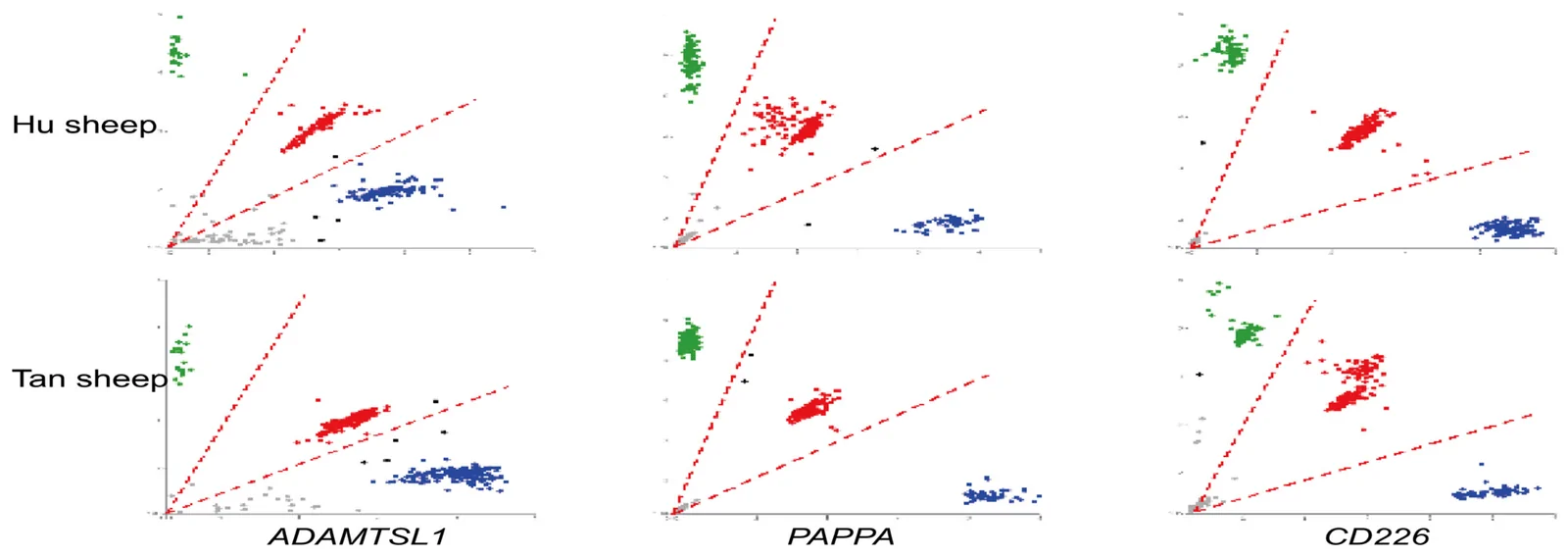
PARMS fluorescence-clustering plots for the three candidate SNPs in Hu and Tan sheep. Colored clusters represent the assigned genotype classes, and dashed lines indicate visual cluster boundaries.

PARMS genotyping produced clearly separated fluorescence clusters for the three candidate SNPs in both populations, with call rates ranging from 98.83% to 100% (Figure 5; Table 7). The genotype distribution of *ADAMTSL1* was similar between Hu and Tan sheep, with GG being the predominant genotype in both populations (57.71% and 59.57%, respectively). For *PAPPA*, GG was the most frequent genotype in Hu sheep (50.00%), whereas AG was the most frequent genotype in Tan sheep (46.81%). In contrast, *CD226* showed a pronounced population difference: AA predominated in Hu sheep (87.11%), whereas AG and GG accounted for 59.57% and 34.04%, respectively, in Tan sheep. Consistent with the genotype distributions, the G allele was predominant at *ADAMTSL1* and *PAPPA* in both populations. At the *CD226* locus, however, the A allele predominated in Hu sheep (92.77%), whereas the G allele predominated in Tan sheep (63.83%) (Table 7). Detailed genotype counts and allele frequencies are provided in Table S1.

### 3.6. Genetic Polymorphism and Hardy–Weinberg Equilibrium

Genetic diversity varied among loci and populations (Table 7). *ADAMTSL1* exhibited moderate polymorphism in Hu and Tan sheep, with PIC values of 0.29 and 0.29, respectively. *PAPPA* also showed moderate polymorphism in both populations, with PIC values of 0.33 in Hu sheep and 0.36 in Tan sheep. In contrast, *CD226* showed low polymorphism in Hu sheep (PIC = 0.13) but moderate polymorphism in Tan sheep (PIC = 0.36). Exact Hardy–Weinberg equilibrium tests indicated that *ADAMTSL1* and *PAPPA* were consistent with equilibrium expectations in both populations. However, *CD226* deviated from Hardy–Weinberg equilibrium in Hu sheep (*p* = 0.03) and Tan sheep (*p* = 0.01).

### 3.7. Candidate-SNP Association Analysis

**Figure 6:**
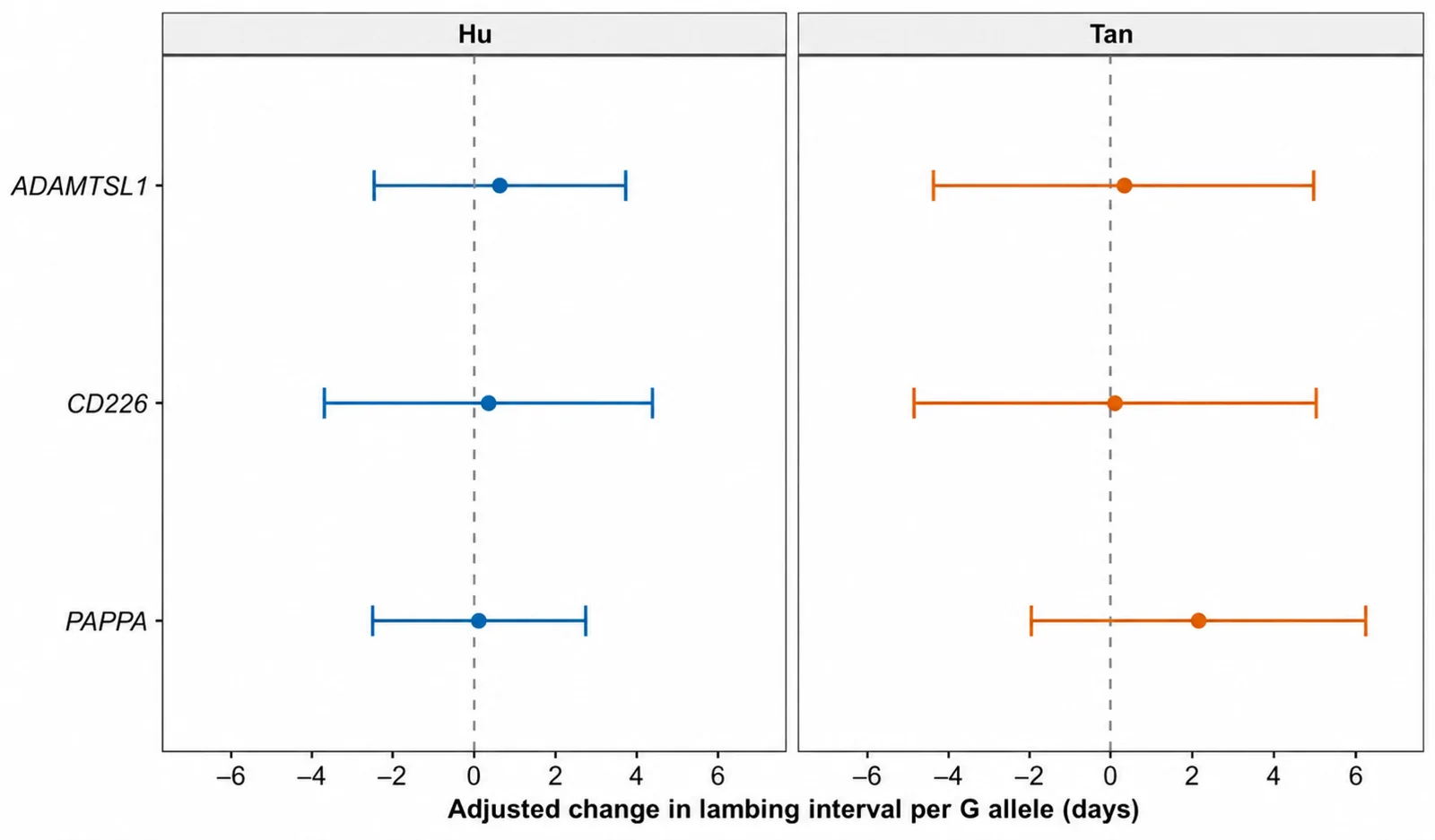
Adjusted additive effects of the three candidate SNPs on lambing interval in Hu and Tan sheep. Points show the estimated change in lambing interval per additional G allele, and horizontal bars show the corresponding 95% confidence intervals, estimated from population-specific linear mixed models adjusted for parity group, year and season of the preceding lambing, and preceding litter size, with ewe identity fitted as a random intercept. The vertical dashed line indicates no effect.

None of the three candidate SNPs showed a significant additive association with lambing interval in either population (Figure 6; Table 8). In Hu sheep, the adjusted effects per additional G allele were 0.63 days for *ADAMTSL1*, 0.36 days for *CD226*, and 0.12 days for *PAPPA*. The corresponding effects in Tan sheep were 0.31, 0.09, and 2.16 days, respectively. All 95% confidence intervals included zero, and none of the six population-specific tests remained significant after FDR correction. Sensitivity analyses combining rare genotype classes yielded consistent results (Table S2). Analyses using the expanded 180–730-day and 180–900-day ranges were also consistent with the primary findings; none of the six population-specific SNP tests remained significant after FDR correction (Table S4). None of the population-by-SNP interactions were statistically significant after FDR correction (*ADAMTSL1:* interaction β = −1.76 days; *CD226*: β = −0.93 days; *PAPPA:* β = 1.87 days; all FDR-adjusted *p* = 0.781; Table S5).

### 3.8. Association with Within-Ewe Lambing-Interval Variability

Exploratory analyses of within-ewe lambing-interval variability were restricted to ewes with at least three valid lambing intervals, including 142 Hu ewes and 39 Tan ewes. None of the candidate SNPs showed a significant additive association with interval SD, MAD, or CV after FDR correction. In Hu sheep, minor-allele carriers at *CD226* showed a tendency toward lower interval variability, but the comparisons of AG+GG versus AA were not significant for SD, MAD, or CV, and the permutation tests provided no statistical support (Table S3).

## 4. Discussion

Lambing interval reflects the continuity of reproductive performance across successive parities and provides information complementary to litter size. In the present study, we characterized population-level lambing-interval patterns and evaluated three previously reported candidate SNPs in Hu and Tan sheep.

A pronounced population-level difference in lambing interval was observed, with Hu sheep showing a substantially shorter adjusted interval than Tan sheep. The direction of this difference remained consistent across parity groups and in the winter-matched analysis. Sensitivity analyses using expanded ranges of 180–730 and 180–900 days also retained the same direction and statistical significance, although inclusion of very long intervals increased the estimated magnitude of the population difference. These findings indicate that the observed contrast was not created solely by the original 450-day upper threshold. Nevertheless, because population, breed, farm, geographic region, and production environment were confounded, the difference cannot be attributed exclusively to genetic background. Reproductive seasonality, mating schedules, nutritional conditions, health status, and other management factors may also have contributed. The parity-specific estimates require particular caution in the later-parity groups. Only 28 Tan intervals contributed to the 4+ group, compared with 71 in the 1–2 group, and ewes reaching a fourth or later lambing are by definition those that remained reproductively active and were retained in the flock. The 4+ estimate may therefore be influenced both by reduced precision and by selection for reproductively persistent ewes, and it should not be read as evidence that the population difference widens with parity.

*PAPPA* encodes pregnancy-associated plasma protein-A, a metalloproteinase that regulates local insulin-like growth factor (IGF) bioavailability by cleaving IGF-binding proteins. Experimental loss or reduction in PAPP-A activity has been reported to impair ovarian steroidogenesis and female reproductive function [14]. Evidence from the broader pappalysin family also suggests a potential role in livestock reproductive traits. For example, variants in the related *PAPPA*2 gene have been associated with litter size in sheep and with prolonged rebreeding intervals and delayed calving in cattle, providing indirect support for the involvement of this pathway in reproductive timing [29,30]. In the present study populations, the tested *PAPPA* SNP showed moderate polymorphism, but no significant association was detected with adjusted lambing interval or within-ewe interval variability. These findings do not support a detectable effect of this specific SNP on lambing interval in the studied Hu and Tan sheep populations. Nevertheless, they do not exclude the possibility that other functional or linked variants within or near *PAPPA* may contribute to reproductive timing and warrant further investigation.

*ADAMTSL1* belongs to the ADAMTS-like family of secreted glycoproteins, which lack protease activity but bind fibrillin microfibrils and help regulate extracellular-matrix organization and transforming growth factor-beta signaling during tissue remodeling [19]. These functions provide a plausible, although indirect, biological link to reproductive tissues, because postpartum uterine involution, follicular development, and preparation for subsequent pregnancy involve extensive extracellular-matrix remodeling. However, the proteolytic cleavage of versican during follicular and cumulus remodeling is mediated by catalytically active ADAMTS proteins rather than by *ADAMTSL1* itself. Notably, a genome-wide association study in Lori-Bakhtiari ewes identified *ADAMTSL1* rs415581052, originally reported as s50067.1, as being significantly associated with lambing interval [22]. In the present study populations, the tested *ADAMTSL*1 SNP showed moderate polymorphism, with the G allele predominating in both populations; however, the variant was not significantly associated with adjusted lambing interval or within-ewe interval variability. The contrast with the reported GWAS signal more likely reflects causal variation elsewhere in or near the gene, or an effect too small to detect with a single marker in a sample of this size, than an absence of any role for *ADAMTSL1*.

Of the three loci, *CD226* displayed the most distinct population-specific frequency pattern. It segregated as an A/G polymorphism in both Hu and Tan sheep, but showed marked differences in genotype and allele frequencies between the two study populations. *CD226* is an activating immune receptor on T and natural killer cells that, together with its inhibitory counterpart TIGIT, helps set the balance between immune activation and tolerance; this checkpoint axis operates at the maternal–fetal interface and has been examined in the context of pregnancy and pregnancy disorders such as preeclampsia [31]. These immunological functions provide a possible, but indirect, biological rationale for examining *CD226* in reproductive traits. However, the tested SNP showed no significant association with adjusted lambing interval or within-ewe interval variability in either population. Moreover, the locus deviated from Hardy–Weinberg equilibrium in both populations, which warrants additional caution. Therefore, the observed frequency differences should not be interpreted as evidence that this SNP contributes to the population-level difference in reproductive rhythm. They may instead reflect differences in genetic background, breeding history, population structure, selection, sampling, or residual genotyping uncertainty.

Among the three tested variants, only *CD226* rs404360094 is a missense variant with a direct protein-coding consequence. In contrast, *ADAMTSL1* rs415581052 and *PAPPA* rs420485231 are intronic variants and should be regarded as previously reported marker loci rather than established functional mutations. Associations involving these intronic variants may arise through linkage disequilibrium with regulatory or causal variants located elsewhere within or near the corresponding genomic regions. Because gene-expression, regulatory, and protein-function assays were not performed, the biological consequences of the three variants remain unresolved.

None of the three candidate SNPs showed a statistically supported additive association with lambing interval after FDR correction. Exploratory analyses of within-ewe lambing-interval variability also yielded no significant associations. Although *CD226* G-allele carriers in Hu sheep showed a directional tendency toward lower SD, MAD, and CV, this pattern was not supported by FDR correction or permutation testing. Therefore, the present findings do not support the use of any tested SNP as an independent marker for lambing interval or within-ewe variability. More broadly, the results are consistent with lambing interval being a complex longitudinal phenotype influenced by multiple genetic, environmental, and management factors rather than by a single large-effect locus.

Several limitations should be acknowledged. Some minor genotype classes contained few animals, reducing statistical power and increasing uncertainty in genotype-effect estimates. The within-ewe variability analysis was restricted to ewes with at least three valid intervals, resulting in a particularly small Tan sheep sample. Only three candidate SNPs were investigated, and genome-wide genotype data were unavailable; therefore, population structure, genomic relatedness, local linkage disequilibrium, and haplotype effects could not be evaluated. The lambing interval was calculated from consecutive recorded lambing dates rather than from complete reproductive-event histories. Because information on ram exposure, mating opportunities, conception, pregnancy diagnosis, abortion, pregnancy loss, skipped breeding seasons, and missed lambing events was not consistently available, prolonged intervals may represent a combination of biological reproductive performance, management practices, and recording completeness. Finally, the two populations originated from different farms and environments, and no functional validation was conducted. Larger independent and multi-farm populations, together with genome-wide, haplotype-based, and functional analyses, are therefore required to clarify the contribution of these genomic regions to reproductive timing in sheep.

## 5. Conclusions

This study revealed a marked population-level difference in lambing interval between Hu and Tan sheep under their respective production environments, with Hu sheep showing a substantially shorter adjusted interval. None of the three candidate SNPs in *ADAMTSL1*, *CD226*, and *PAPPA* was significantly associated with lambing interval after FDR correction. Although *CD226* G-allele carriers in Hu sheep showed a weak tendency toward lower within-ewe lambing-interval variability, this pattern was not supported by FDR correction or permutation testing. Therefore, the tested SNPs cannot currently be recommended as independent markers for lambing interval or within-ewe lambing-interval variability. Validation in larger, independent, multi-farm populations using genome-wide, haplotype-based, and functional analyses is warranted.

## Figures and Tables

**Table 1:** Definitions of lambing interval and within-ewe variability metrics.

Metric	Definition
Mean interval	Mean value of all valid lambing intervals for each ewe
Interval SD	Standard deviation of valid lambing intervals for each ewe
Interval CV	Interval SD divided by mean interval
Interval MAD	Scaled median absolute deviation of valid lambing intervals within each ewe

Note: SD, standard deviation; MAD, scaled median absolute deviation; CV, coefficient of variation. These metrics were used as descriptive and exploratory measures of within-ewe lambing-interval variability rather than as validated biological stability traits or predictors of future reproductive success.

**Table 2:** Primers used for PCR amplification and Sanger sequencing validation of candidate SNPs.

Gene	Chr:bp	SNP	Alleles	Genomic Region	Predicted Consequence	Primer Sequences (5′–3′)	Ta	Size	References
*ADAMTSL1*	2:87396795	rs415581052	A/G	Intron	Intron variant	F:GGACCATCACTCCCCATCTG R:AAGGCACAATGACAGAGACACA	55	456	[22]
*PAPPA*	2:7400929	rs420485231	G/A	Intron	Intron variant	F:TGTTGGAAAGTCGGGGAGC R:GGGATACAGAGTGGGGATGAGT	54	483	[15]
*CD226*	23:7578216	rs404360094	A/G	Exon	Missense variant	F:ACATGTGCATTGCGGATCAAT R:AAACTGGACTGAAGGCCAAGG	57	533	[18]

Note: Genomic positions are based on the *Ovis aries* ARS-UI_Ramb_v3.0 reference genome assembly.

**Table 3:** Primer sequences used for PARMS-based genotyping of SNPs in the *ADAMTSL1*, *PAPPA*, and *CD226* genes.

Gene	SNP	Primer Name	Primer Sequence (5′–3′)
*ADAMTSL1*	rs415581052	*ADAMTSL*1-Rc	GAAGGTGACCAAGTTCATGCTGGGTCAAGTTGGAGACATGGC
*ADAMTSL1*	rs415581052	*ADAMTSL1*-Rt	GAAGGTCGGAGTCAACGGATTTGGGTCAAGTTGGAGACATGGT
*ADAMTSL1*	rs415581052	*ADAMTSL1*-F	AACAGCTTTGGTCAGCTCTTCTG
*PAPPA*	rs420485231	*PAPPA*-Rt	GAAGGTGACCAAGTTCATGCTCAGAGGGGCAACATTAGAGAGAAT
*PAPPA*	rs420485231	*PAPPA*-Rc	GAAGGTCGGAGTCAACGGATTCAGAGGGGCAACATTAGAGAGAAC
*PAPPA*	rs420485231	*PAPPA*-F	GGCAGTGTCTTGCAACAGCTC
*CD226*	rs404360094	*CD226*-Fc	GAAGGTGACCAAGTTCATGCTGTGGTGATTTGGGATGCAAAC
*CD226*	rs404360094	*CD226*-Fa	GAAGGTCGGAGTCAACGGATTGTGGTGATTTGGGATGCAAAA
*CD226*	rs404360094	*CD226*-R	CTTACTTGCTTTTTCTAAACCATCTTC

**Table 4:** Adjusted lambing intervals and population contrasts in the main and winter-matched analyses.

Analysis	Hu Sheep Adjusted Mean, Days (95% CI)	Tan Sheep Adjusted Mean, Days (95% CI)	Tan−Hu Difference, Days	95% CI	*p* Value
Main analysis	288.31 (281.83–294.80)	369.30 (361.36–377.25)	80.99	75.34–86.64	3.17 × 10^−126^
Winter-matched analysis	289.92 (283.20–296.64)	371.57 (367.09–376.04)	81.65	73.37–89.93	1.88 × 10^−56^

**Table 5:** Adjusted lambing intervals and population contrasts across parity groups.

Parity Group	Hu Sheep	Hu Sheep 95% CI	Tan Sheep	Tan Sheep 95% CI	Difference, Days	SE	95% CI	*p* Value	FDR-Adjusted *p* Value
1–2	289.71	282.99–296.43	369.32	360.00–378.63	79.61	3.99	71.78–87.43	2.61 × 10^−74^	5.23 × 10^−74^
2–3	287.11	279.98–294.23	372.59	363.31–381.87	85.48	4.10	77.44–93.52	6.93 × 10^−80^	2.77 × 10^−79^
3–4	289.32	281.82–296.81	362.05	351.73–372.37	72.73	4.71	63.49–81.98	3.63 × 10^−48^	3.63 × 10^−48^
4+	287.18	279.75–294.61	374.11	362.45–385.77	86.93	5.46	76.23–97.64	9.60 × 10^−51^	1.28 × 10^−50^

**Table 6:** Fixed effects from the linear mixed model for lambing interval.

Fixed Effect	Numerator df	Denominator df	F Value	*p* Value
Population	1	930	745.538	5.08 × 10^−121^
Parity group	3	930	1.181	0.316
Year	7	930	4.863	2.12 × 10^−5^
Season of preceding lambing	3	930	59.71	2.63 × 10^−35^
Preceding litter size	1	930	0.008	0.928
Population × parity group	3	930	2.721	0.043

**Table 7:** Genotype distribution, genetic diversity, call rate, and exact Hardy–Weinberg equilibrium tests for the candidate SNPs.

Population	Gene	N	Call Rate (%)	AA	AG	GG	A	G	Ho	He	Ne	PIC	HWE *p*
Hu sheep	*ADAMTSL1*	253	98.83	8	99	146	0.23	0.77	0.39	0.35	1.54	0.29	0.11
Tan sheep	*ADAMTSL1*	94	100	4	34	56	0.22	0.78	0.36	0.35	1.53	0.29	1.00
Hu sheep	*CD226*	256	100	223	29	4	0.93	0.07	0.11	0.13	1.16	0.13	0.03
Tan sheep	*CD226*	94	100	6	56	32	0.36	0.64	0.60	0.46	1.86	0.36	0.01
Hu sheep	*PAPPA*	256	100	27	101	128	0.30	0.70	0.40	0.42	1.73	0.33	0.30
Tan sheep	*PAPPA*	94	100	12	44	38	0.36	0.64	0.47	0.46	1.86	0.36	1.00

Note: Ho, observed heterozygosity; He, expected heterozygosity; Ne, effective number of alleles; PIC, polymorphism information content; HWE *p*, *p* value from the exact Hardy–Weinberg equilibrium test.

**Table 8:** Adjusted additive effects of the three candidate SNPs on lambing interval.

Population	Gene	Ewes	Intervals	β	SE	95% CI	*p*	FDR
Hu sheep	*ADAMTSL1*	228	704	0.63	1.59	−2.50–3.75	0.693	0.971
Tan sheep	*ADAMTSL1*	82	219	0.31	2.38	−4.35–4.97	0.897	0.971
Hu sheep	*CD226*	230	711	0.36	2.07	−3.69–4.41	0.863	0.971
Tan sheep	*CD226*	82	219	0.09	2.53	−4.86–5.04	0.971	0.971
Hu sheep	*PAPPA*	230	711	0.12	1.35	−2.53–2.77	0.930	0.971
Tan sheep	*PAPPA*	82	219	2.16	2.1	−1.96–6.27	0.306	0.971

Note: *β*, estimated change in lambing interval per additional G allele; SE, standard error; CI, confidence interval; FDR, Benjamini–Hochberg false-discovery-rate-adjusted *p* value.

## Data Availability

The phenotype and genotype data generated and analyzed in this study are available from the corresponding author upon reasonable request, subject to institutional and farm-record restrictions.

## Supplementary Materials

The following supporting information can be downloaded at https://www.mdpi.com/article/10.3390/ani16162527/s1, Table S1: Genotype counts among ewes included in the phenotype association analyses; Table S2: Combined-category sensitivity analyses for rare genotype classes; Table S3: Exploratory associations between candidate SNPs and within-ewe lambing interval variability; Table S4: Sensitivity analyses of population contrasts and candidate-SNP associations using expanded lambing-interval ranges; Table S5: Population-by-SNP interaction analyses and AR(1) repeated-measures sensitivity analysis; File S1: Annotated R script for data preparation, statistical analyses, sensitivity analyses, permutation testing, genetic-diversity summaries, and generation of the final R-generated manuscript figures.
