# Supplementary material for: Population-Level Lambing Interval Patterns and Evaluation of Three Previously Reported Candidate SNPs in Hu and Tan Sheep
Source: Animals (Basel). 2026 Aug 13;16(16):2527. doi: 10.3390/ani16162527 (PMC13508885; doi:10.3390/ani16162527)
Supplement: Supplementary file 1 [file animals-16-02527-s001.zip › S1.pdf]

```
options(stringsAsFactors = FALSE, scipen = 999)
set.seed(20260713)
args <- commandArgs(trailingOnly = TRUE)
project_dir <- if (length(args) >= 1) args[1] else getwd()
project_dir <- normalizePath(project_dir, winslash = "/", mustWork = FALSE)
resolve_input_file <- function(candidates, label) {
  hits <- candidates[file.exists(candidates)]
  if (length(hits) == 0) {
    stop(
      "Unable to locate the ", label, " workbook. Checked: ",
      paste(candidates, collapse = "; ")
    )
  }
  hits[1]
}

hu_file <- resolve_input_file(
  c(
    file.path(project_dir, "Hu_sheep.xlsx"),
    file.path(project_dir, "\u6E56\u7F8A.xlsx")
  ),
  "Hu sheep"
)
tan_file <- resolve_input_file(
  c(
    file.path(project_dir, "Tan_sheep.xlsx"),
    file.path(project_dir, "\u6EE9\u7F8A.xlsx")
  ),
  "Tan sheep"
)
out_dir    <- file.path(project_dir, "reanalysis_results_1000dpi")
fig_dir    <- file.path(out_dir, "figures")
table_dir <- file.path(out_dir, "tables")
data_dir   <- file.path(out_dir, "clean_data")
model_dir <- file.path(out_dir, "models")
dir.create(out_dir,    recursive = TRUE, showWarnings = FALSE)
dir.create(fig_dir,    recursive = TRUE, showWarnings = FALSE)
dir.create(table_dir, recursive = TRUE, showWarnings = FALSE)
dir.create(data_dir,   recursive = TRUE, showWarnings = FALSE)
dir.create(model_dir, recursive = TRUE, showWarnings = FALSE)
required_packages <- c(
  "readxl", "dplyr", "tidyr", "stringr", "lubridate", "purrr",
  "ggplot2", "patchwork", "lme4", "lmerTest", "emmeans",
  "writexl", "HardyWeinberg", "forcats", "scales", "ragg"
```

```
)
not_installed <- setdiff(required_packages, rownames(installed.packages()))
if (length(not_installed) > 0) {
  install.packages(not_installed, dependencies = TRUE)
}
invisible(lapply(required_packages, library, character.only = TRUE))
options(contrasts = c("contr.treatment", "contr.poly"))
theme_publication <- function(base_size = 11) {
  theme_classic(base_size = base_size, base_family = "sans") +
    theme(
      axis.title = element_text(size = base_size + 1, face = "bold"),
      axis.text = element_text(size = base_size, colour = "black"),
      strip.background = element_rect(fill = "grey92", colour = "black", linewidth = 0.5),
      strip.text = element_text(size = base_size, face = "bold"),
      legend.title = element_text(face = "bold"),
      legend.text = element_text(size = base_size - 1),
      plot.title = element_text(size = base_size + 2, face = "bold", hjust = 0),
      plot.subtitle = element_text(size = base_size),
      plot.tag = element_text(size = base_size + 2, face = "bold"),
      panel.border = element_rect(fill = NA, colour = "black", linewidth = 0.5),
      axis.line = element_blank(),
      plot.margin = margin(8, 10, 8, 8)
    )
}
theme_set(theme_publication())
population_cols <- c("Hu" = "#2C7FB8", "Tan" = "#D95F0E")
genotype_cols <- c("AA" = "#4C78A8", "AG" = "#F2CF5B", "GG" = "#E45756")
allele_cols <- c("A" = "#4C78A8", "G" = "#E45756")

save_pub <- function(plot_object, file_stub, width = 8.5, height = 6.5) {
  tiff_file <- file.path(fig_dir, paste0(file_stub, ".tiff"))
  png_file  <- file.path(fig_dir, paste0(file_stub, ".png"))
  pdf_file  <- file.path(fig_dir, paste0(file_stub, ".pdf"))
  ragg::agg_tiff(
    filename = tiff_file,
    width = width, height = height, units = "in",
    res = 1000, compression = "lzw", background = "white"
  )
  print(plot_object)
  dev.off()
  ragg::agg_png(
    filename = png_file,
    width = width, height = height, units = "in",
    res = 1000, background = "white"
```

```r
    )
    print(plot_object)
    dev.off()
    ggsave(
        filename = pdf_file, plot = plot_object,
        width = width, height = height, units = "in",
        device = grDevices::cairo_pdf, bg = "white"
    )
}
clean_id <- function(x) {
    x <- stringr::str_squish(as.character(x))
    x <- stringr::str_replace(x, "\\.0$", "")
    x[x %in% c("", "NA", "N/A", "NULL")] <- NA_character_
    toupper(x)
}
normalize_diploid <- function(x) {
    z <- toupper(stringr::str_squish(as.character(x)))
    z <- stringr::str_replace_all(z, "[^A-Z]", "")
    z[z %in% c("", "NA", "NAN", "NULL")] <- NA_character_
    vapply(z, function(g) {
        if (is.na(g) || nchar(g) != 2) return(NA_character_)
        paste(sort(strsplit(g, "")[[1]]), collapse = "")
    }, FUN.VALUE = character(1))
}
normalize_cd226_hu <- function(x) {
    z <- normalize_diploid(x)
    dplyr::recode(z, "AT" = "AG", "TT" = "GG", .default = z)
}
parse_excel_date <- function(x) {
    if (inherits(x, "Date")) return(as.Date(x))
    if (inherits(x, "POSIXt")) return(as.Date(x))
    x_chr <- stringr::str_squish(as.character(x))
    x_chr[x_chr %in% c("", "NA", "N/A", "NULL")] <- NA_character_
    x_num <- suppressWarnings(as.numeric(x_chr))
    out <- as.Date(rep(NA_character_, length(x_chr)))
    idx_num <- !is.na(x_num)
    if (any(idx_num)) {
        out[idx_num] <- as.Date(x_num[idx_num], origin = "1899-12-30")
    }
    idx_txt <- !idx_num & !is.na(x_chr)
    if (any(idx_txt)) {
        parsed <- suppressWarnings(
            lubridate::parse_date_time(
                x_chr[idx_txt],
```

```
        orders = c("ymd", "Ymd", "Y/m/d", "Y-m-d", "mdy", "dmy")
      )
    )
    out[idx_txt] <- as.Date(parsed)
  }
  out
}
season_from_month <- function(month_num) {
  factor(
    dplyr::case_when(
      month_num %in% c(12, 1, 2) ~ "Winter",
      month_num %in% c(3, 4, 5)    ~ "Spring",
      month_num %in% c(6, 7, 8)    ~ "Summer",
      month_num %in% c(9, 10, 11) ~ "Autumn",
      TRUE ~ NA_character_
    ),
    levels = c("Winter", "Spring", "Summer", "Autumn")
  )
}
gt_dosage_G <- function(gt) {
  gt <- normalize_diploid(gt)
  ifelse(is.na(gt), NA_real_, stringr::str_count(gt, "G"))
}
safe_lmer <- function(formula, data) {
  tryCatch(
    lmerTest::lmer(
      formula = formula,
      data = droplevels(data),
      REML = FALSE,
      control = lme4::lmerControl(
        optimizer = "bobyqa",
        optCtrl = list(maxfun = 200000)
      )
    ),
    error = function(e) {
      message("Model fitting failed: ", conditionMessage(e))
      return(NULL)
    }
  )
}
extract_lmer_term <- function(model, term_name) {
  if (is.null(model)) {
    return(data.frame(
      estimate = NA_real_, se = NA_real_, statistic = NA_real_,
```

```
            df = NA_real_, p_value = NA_real_, conf_low = NA_real_,
            conf_high = NA_real_
        ))
    }
    sm <- coef(summary(model))
    if (!term_name %in% rownames(sm)) {
        return(data.frame(
            estimate = NA_real_, se = NA_real_, statistic = NA_real_,
            df = NA_real_, p_value = NA_real_, conf_low = NA_real_,
            conf_high = NA_real_
        ))
    }
    ci <- suppressMessages(confint(model, parm = term_name, method = "Wald"))
    data.frame(
        estimate = unname(sm[term_name, "Estimate"]),
        se = unname(sm[term_name, "Std. Error"]),
        statistic = unname(sm[term_name, "t value"]),
        df = if ("df" %in% colnames(sm)) unname(sm[term_name, "df"]) else NA_real_,
        p_value = if ("Pr(>|t|)" %in% colnames(sm)) unname(sm[term_name, "Pr(>|t|)"]) else
NA_real_,
        conf_low = unname(ci[1]),
        conf_high = unname(ci[2])
    )
}
extract_lm_term <- function(model, term_name) {
    if (is.null(model)) {
        return(data.frame(
            estimate = NA_real_, se = NA_real_, statistic = NA_real_,
            df = NA_real_, p_value = NA_real_, conf_low = NA_real_,
            conf_high = NA_real_
        ))
    }
    sm <- coef(summary(model))
    if (!term_name %in% rownames(sm)) {
        return(data.frame(
            estimate = NA_real_, se = NA_real_, statistic = NA_real_,
            df = NA_real_, p_value = NA_real_, conf_low = NA_real_,
            conf_high = NA_real_
        ))
    }
    ci <- confint(model, parm = term_name)
    data.frame(
        estimate = unname(sm[term_name, "Estimate"]),
        se = unname(sm[term_name, "Std. Error"]),
```

```r
        statistic = unname(sm[term_name, "t value"]),
        df = unname(df.residual(model)),
        p_value = unname(sm[term_name, "Pr(>|t|)"]),
        conf_low = unname(ci[1]),
        conf_high = unname(ci[2])
    )
}
hu_gt_raw <- readxl::read_excel(
    hu_file, sheet = "\u5BBD\u8868_\u8865\u9F50\u540E", .name_repair = "unique"
)
tan_gt_raw <- readxl::read_excel(
    tan_file, sheet = "\u6EE9\u7F8A\u5BBD\u8868", .name_repair = "unique"
)
hu_gt <- hu_gt_raw %>%
    transmute(
        population = "Hu",
        ewe_id = clean_id(.data[["\u7F8A\u53F7"]]),
        ADAMTSL1 = normalize_diploid(`ANAMTSL1`),
        CD226 = normalize_cd226_hu(`CD226`),
        PAPPA = normalize_diploid(`PAPPA`)
    ) %>%
    filter(!is.na(ewe_id)) %>%
    distinct(population, ewe_id, .keep_all = TRUE)
tan_gt <- tan_gt_raw %>%
    transmute(
        population = "Tan",
        ewe_id = clean_id(.data[["\u6BCD\u7F8A\u8033\u53F7"]]),
        ADAMTSL1 = normalize_diploid(`adamtsl1`),
        CD226 = normalize_diploid(`CD226`),
        PAPPA = normalize_diploid(`pappa`)
    ) %>%
    filter(!is.na(ewe_id)) %>%
    distinct(population, ewe_id, .keep_all = TRUE)
gt_all <- bind_rows(hu_gt, tan_gt) %>%
    mutate(population = factor(population, levels = c("Hu", "Tan")))
hu_lamb_raw <- readxl::read_excel(
    hu_file, sheet = "\u957F\u8868_\u4EA7\u7F94\u8BB0\u5F55", .name_repair = "unique"
)
tan_lamb_raw <- readxl::read_excel(
    tan_file, sheet = "\u4EA7\u7F94\u957F\u8868", .name_repair = "unique"
)
hu_lamb <- hu_lamb_raw %>%
    transmute(
        population = "Hu",
```

```
    ewe_id = clean_id(.data[["\u7F8A\u53F7"]]),
    parity = suppressWarnings(as.integer(.data[["\u80CE\u6B21"]])),
    raw_lambing_date = as.character(.data[["\u4EA7\u7F94\u65E5\u671F"]]),
    lambing_date = parse_excel_date(.data[["\u4EA7\u7F94\u65E5\u671F"]]),
    litter_size = suppressWarnings(as.numeric(.data[["\u4EA7\u7F94\u6570_\u603B\u7F94"]])),
    data_source = as.character(.data[["\u6570\u636E\u6765\u6E90"]])
  )
tan_lamb <- tan_lamb_raw %>%
  transmute(
    population = "Tan",
    ewe_id = clean_id(.data[["\u6BCD\u7F8A\u8033\u53F7"]]),
    parity                                                                        =
suppressWarnings(as.integer(stringr::str_extract(as.character(.data[["\u80CE\u6B21"]]),
"\\d+"))),
    raw_lambing_date = as.character(.data[["\u4EA7\u7F94\u65E5\u671F"]]),
    lambing_date = parse_excel_date(.data[["\u4EA7\u7F94\u65E5\u671F"]]),
    litter_size = suppressWarnings(as.numeric(.data[["\u4EA7\u7F94\u6570"]])),
    data_source = paste0(basename(tan_file), "::\u4EA7\u7F94\u957F\u8868")
  )
lambing_all <- bind_rows(hu_lamb, tan_lamb) %>%
  mutate(
    population = factor(population, levels = c("Hu", "Tan")),
    min_allowed_date = as.Date("2010-01-01"),
    max_allowed_date = as.Date("2030-12-31"),
    date_valid = !is.na(lambing_date) &
      lambing_date >= min_allowed_date &
      lambing_date <= max_allowed_date,
    litter_size_valid = is.na(litter_size) | (litter_size >= 1 & litter_size <= 8),
    lambing_date_clean = if_else(date_valid, lambing_date, as.Date(NA)),
    litter_size_clean = if_else(litter_size_valid, litter_size, NA_real_)
  ) %>%
  select(-min_allowed_date, -max_allowed_date)
date_anomalies <- lambing_all %>%
  filter(!date_valid | !litter_size_valid | is.na(ewe_id) | is.na(parity)) %>%
  arrange(population, ewe_id, parity)
duplicate_parity_records <- lambing_all %>%
  filter(!is.na(ewe_id), !is.na(parity)) %>%
  count(population, ewe_id, parity, name = "n_records") %>%
  filter(n_records > 1)
lambing_clean <- lambing_all %>%
  filter(!is.na(ewe_id), !is.na(parity)) %>%
  arrange(population, ewe_id, parity, desc(date_valid), lambing_date_clean) %>%
  group_by(population, ewe_id, parity) %>%
  slice(1) %>%
```

```
  ungroup() %>%
  select(
    population, ewe_id, parity, raw_lambing_date,
    lambing_date = lambing_date_clean,
    litter_size = litter_size_clean,
    date_valid, litter_size_valid, data_source
  )
interval_all <- lambing_clean %>%
  group_by(population, ewe_id) %>%
  arrange(parity, .by_group = TRUE) %>%
  mutate(
    next_parity = lead(parity),
    next_lambing_date = lead(lambing_date),
    next_litter_size = lead(litter_size),
    adjacent_parity = !is.na(next_parity) & next_parity == parity + 1,
    interval_days = as.integer(next_lambing_date - lambing_date)
  ) %>%
  ungroup() %>%
  filter(!is.na(next_parity)) %>%
  mutate(
    interval_status = case_when(
      !adjacent_parity ~ "Non-adjacent parity",
      is.na(lambing_date) | is.na(next_lambing_date) ~ "Invalid/missing date",
      is.na(interval_days) ~ "Missing interval",
      interval_days < 180 ~ "<180 d",
      interval_days > 450 ~ ">450 d",
      TRUE ~ "Valid 180-450 d"
    ),
    valid_180_450 = interval_status == "Valid 180-450 d",
    parity_interval = paste0(parity, "-", next_parity),
    parity_group = case_when(
      parity == 1 ~ "1-2",
      parity == 2 ~ "2-3",
      parity == 3 ~ "3-4",
      parity >= 4 ~ "4+",
      TRUE ~ NA_character_
    ),
    parity_group = factor(parity_group, levels = c("1-2", "2-3", "3-4", "4+")),
    prev_year = year(lambing_date),
    prev_month = month(lambing_date),
    prev_season = season_from_month(prev_month),
    ewe_uid = paste(population, ewe_id, sep = "_")
  ) %>%
  left_join(gt_all, by = c("population", "ewe_id"))
```

```
valid_intervals <- interval_all %>%
  filter(valid_180_450) %>%
  mutate(
    population = factor(population, levels = c("Hu", "Tan")),
    prev_year_f = factor(prev_year),
    prev_season = factor(
      prev_season,
      levels = c("Winter", "Spring", "Summer", "Autumn")
    )
  )
n_with_two_lambings <- lambing_clean %>%
  filter(!is.na(lambing_date)) %>%
  count(population, ewe_id, name = "n_lambing") %>%
  filter(n_lambing >= 2) %>%
  count(population, name = "ewes_with_2plus_lambings")
candidate_intervals <- interval_all %>%
  filter(adjacent_parity, !is.na(interval_days)) %>%
  count(population, name = "candidate_intervals")
qc_flow <- gt_all %>%
  count(population, name = "genotyped_ewes") %>%
  left_join(n_with_two_lambings, by = "population") %>%
  left_join(candidate_intervals, by = "population") %>%
  left_join(
    valid_intervals %>%
      summarise(
        valid_intervals = n(),
        valid_ewes = n_distinct(ewe_id),
        .by = population
      ),
    by = "population"
  )
invalid_reason_summary <- interval_all %>%
  count(population, interval_status, name = "n") %>%
  arrange(population, desc(n))
overall_summary <- valid_intervals %>%
  group_by(population) %>%
  summarise(
    n_ewes = n_distinct(ewe_id),
    n_intervals = n(),
    mean_days = mean(interval_days),
    sd_days = sd(interval_days),
    median_days = median(interval_days),
    iqr_days = IQR(interval_days),
    min_days = min(interval_days),
```

```
    max_days = max(interval_days),
    .groups = "drop"
  )
parity_summary <- valid_intervals %>%
  group_by(population, parity_group) %>%
  summarise(
    n_ewes = n_distinct(ewe_id),
    n_intervals = n(),
    mean_days = mean(interval_days),
    sd_days = sd(interval_days),
    se_days = sd_days / sqrt(n_intervals),
    ci_low = mean_days - qt(0.975, pmax(n_intervals - 1, 1)) * se_days,
    ci_high = mean_days + qt(0.975, pmax(n_intervals - 1, 1)) * se_days,
    min_days = min(interval_days),
    max_days = max(interval_days),
    .groups = "drop"
  )
main_model_data <- valid_intervals %>%
  filter(
    !is.na(interval_days),
    !is.na(parity_group),
    !is.na(prev_year_f),
    !is.na(prev_season),
    !is.na(litter_size),
    !is.na(ewe_uid)
  ) %>%
  mutate(
    population = factor(population, levels = c("Hu", "Tan")),
    ewe_uid = factor(ewe_uid)
  )
main_model <- lmerTest::lmer(
  interval_days ~ population + parity_group + prev_year_f +
    prev_season + litter_size + (1 | ewe_uid),
  data = droplevels(main_model_data),
  REML = FALSE,
  control = lme4::lmerControl(
    optimizer = "bobyqa",
    optCtrl = list(maxfun = 200000)
  )
)
main_population_effect <- extract_lmer_term(main_model, "populationTan") %>%
  mutate(
    analysis = "Main unified QC",
    interpretation = "Tan minus Hu, adjusted difference in days"
```

```r
  )
main_emmeans <- if (!is.null(main_model)) {
  as.data.frame(emmeans::emmeans(main_model, ~ population)) %>%
    transmute(
      population,
      emmean,
      SE,
      df,
      lower.CL,
      upper.CL,
      analysis = "Main: unified 180-450 d"
    )
} else {
  data.frame()
}
hu_years <- sort(unique(valid_intervals$prev_year[valid_intervals$population == "Hu"]))
tan_years <- sort(unique(valid_intervals$prev_year[valid_intervals$population == "Tan"]))
common_years <- intersect(hu_years, tan_years)
sensitivity_data <- valid_intervals %>%
  filter(
    prev_month %in% c(12, 1, 2),
    prev_year %in% common_years,
    !is.na(parity_group),
    !is.na(litter_size)
  ) %>%
  mutate(
    prev_year_f = factor(prev_year),
    population = factor(population, levels = c("Hu", "Tan")),
    ewe_uid = factor(ewe_uid)
  )
sensitivity_model <- lmerTest::lmer(
  interval_days ~ population + parity_group + prev_year_f +
    litter_size + (1 | ewe_uid),
  data = droplevels(sensitivity_data),
  REML = FALSE,
  control = lme4::lmerControl(
    optimizer = "bobyqa",
    optCtrl = list(maxfun = 200000)
  )
)
sensitivity_population_effect <- extract_lmer_term(
  sensitivity_model, "populationTan"
) %>%
  mutate(
```

```
      analysis = paste0(
        "Winter/common years ",
        ifelse(length(common_years) > 0, min(common_years), NA),
        "-",
        ifelse(length(common_years) > 0, max(common_years), NA)
      ),
      interpretation = "Tan minus Hu, adjusted difference in days"
    )
sensitivity_emmeans <- if (!is.null(sensitivity_model)) {
  as.data.frame(emmeans::emmeans(sensitivity_model, ~ population)) %>%
    transmute(
      population,
      emmean,
      SE,
      df,
      lower.CL,
      upper.CL,
      analysis = paste0(
        "Winter + common years (",
        min(common_years), "-", max(common_years), ")"
      )
    )
} else {
  data.frame()
}

population_effects <- bind_rows(
  main_population_effect,
  sensitivity_population_effect
)

population_emmeans <- bind_rows(
  main_emmeans,
  sensitivity_emmeans
)
parity_model <- lmerTest::lmer(
  interval_days ~ population * parity_group + prev_year_f +
    prev_season + litter_size + (1 | ewe_uid),
  data = droplevels(main_model_data),
  REML = FALSE,
  control = lme4::lmerControl(
    optimizer = "bobyqa",
    optCtrl = list(maxfun = 200000)
  )
```

```
)
parity_emmeans <- if (!is.null(parity_model)) {
  as.data.frame(
    emmeans::emmeans(parity_model, ~ population | parity_group)
  ) %>%
    transmute(
      parity_group,
      population,
      emmean,
      SE,
      df,
      lower.CL,
      upper.CL
    )
} else {
  data.frame()
}
parity_model_anova <- if (!is.null(parity_model)) {
  as.data.frame(anova(parity_model)) %>%
    tibble::rownames_to_column("term")
} else {
  data.frame()
}
genes <- c("ADAMTSL1", "CD226", "PAPPA")
fit_snp_additive <- function(data, breed, gene) {
  dat <- data %>%
    filter(population == breed) %>%
    mutate(
      genotype = .data[[gene]],
      dosage_G = gt_dosage_G(genotype)
    ) %>%
    filter(
      !is.na(dosage_G),
      !is.na(parity_group),
      !is.na(prev_year_f),
      !is.na(prev_season),
      !is.na(litter_size)
    ) %>%
    mutate(ewe_id = factor(ewe_id))
  n_ewe <- n_distinct(dat$ewe_id)
  n_interval <- nrow(dat)
  genotype_counts <- dat %>%
    distinct(ewe_id, genotype) %>%
    count(genotype, name = "n") %>%
```

```r
        arrange(genotype)

    if (n_ewe < 15 || length(unique(dat$dosage_G)) < 2) {
        return(list(
            result = data.frame(
                population = breed, gene = gene,
                effect_allele = "G", n_ewes = n_ewe, n_intervals = n_interval,
                estimate = NA_real_, se = NA_real_, statistic = NA_real_,
                df = NA_real_, p_value = NA_real_,
                conf_low = NA_real_, conf_high = NA_real_
            ),
            genotype_counts = genotype_counts,
            model = NULL
        ))
    }
    fit <- safe_lmer(
        interval_days ~ dosage_G + parity_group + prev_year_f +
            prev_season + litter_size + (1 | ewe_id),
        data = dat
    )
    res <- extract_lmer_term(fit, "dosage_G") %>%
        mutate(
            population = breed,
            gene = gene,
            effect_allele = "G",
            n_ewes = n_ewe,
            n_intervals = n_interval,
            .before = 1
        )
    list(result = res, genotype_counts = genotype_counts, model = fit)
}

snp_fit_list <- purrr::cross_df(list(
    population = c("Hu", "Tan"),
    gene = genes
)) %>%
    mutate(
        fit = purrr::map2(
            population, gene,
            ~ fit_snp_additive(valid_intervals, .x, .y)
        )
    )
snp_additive_results <- snp_fit_list %>%
    transmute(result = purrr::map(fit, "result")) %>%
```

```r
  tidyr::unnest(result) %>%
  mutate(
    p_FDR = p.adjust(p_value, method = "BH"),
    significant_FDR = !is.na(p_FDR) & p_FDR < 0.05
  )
snp_genotype_counts <- snp_fit_list %>%
  transmute(
    population,
    gene,
    genotype_counts = purrr::map(fit, "genotype_counts")
  ) %>%
  tidyr::unnest(genotype_counts)
dominant_specs <- tribble(
  ~population, ~gene,          ~reference_group, ~other_group,
  "Hu",        "CD226",        "AA",             "AG+GG",
  "Hu",        "ADAMTSL1",     "GG",             "AA+AG",
  "Tan",       "ADAMTSL1",     "GG",             "AA+AG"
)
fit_dominant_model <- function(population_name, gene_name, reference_group, other_group) {
  dat <- valid_intervals %>%
    filter(population == population_name) %>%
    mutate(genotype = .data[[gene_name]])
  if (reference_group == "AA") {
    dat <- dat %>%
      mutate(genotype_group = if_else(genotype == "AA", "AA", "AG+GG"))
  } else if (reference_group == "GG") {
    dat <- dat %>%
      mutate(genotype_group = if_else(genotype == "GG", "GG", "AA+AG"))
  } else {
    stop("Undefined reference group")
  }
  dat <- dat %>%
    filter(
      !is.na(genotype),
      !is.na(parity_group),
      !is.na(prev_year_f),
      !is.na(prev_season),
      !is.na(litter_size)
    ) %>%
    mutate(
      genotype_group = factor(
        genotype_group,
        levels = c(reference_group, other_group)
      ),
```

```
      ewe_id = factor(ewe_id)
    )
  fit <- safe_lmer(
    interval_days ~ genotype_group + parity_group + prev_year_f +
      prev_season + litter_size + (1 | ewe_id),
    data = dat
  )
  term_name <- paste0("genotype_group", other_group)
  result <- extract_lmer_term(fit, term_name) %>%
    mutate(
      population = population_name,
      gene = gene_name,
      comparison = paste0(other_group, " vs ", reference_group),
      n_ewes = n_distinct(dat$ewe_id),
      n_intervals = nrow(dat),
      .before = 1
    )
  result
}
dominant_results <- pmap_dfr(
  dominant_specs,
  fit_dominant_model
) %>%
  mutate(p_FDR = p.adjust(p_value, method = "BH"))
ewe_stability <- valid_intervals %>%
  group_by(population, ewe_id) %>%
  summarise(
    n_intervals = n(),
    mean_interval = mean(interval_days),
    sd_interval = sd(interval_days),
    cv_interval = sd_interval / mean_interval,
    mad_interval = mad(interval_days, constant = 1.4826),
    min_interval = min(interval_days),
    max_interval = max(interval_days),
    ADAMTSL1 = first(ADAMTSL1),
    CD226 = first(CD226),
    PAPPA = first(PAPPA),
    .groups = "drop"
  ) %>%
  filter(n_intervals >= 3) %>%
  mutate(population = factor(population, levels = c("Hu", "Tan")))
stability_sample_summary <- ewe_stability %>%
  count(population, name = "n_ewes_with_3plus_intervals")
stability_traits <- c(
```

```
  "sd_interval", "mad_interval", "cv_interval"
)
fit_stability_additive <- function(data, breed, gene, trait) {
  dat <- data %>%
    filter(population == breed) %>%
    mutate(
      genotype = .data[[gene]],
      dosage_G = gt_dosage_G(genotype),
      outcome = .data[[trait]]
    ) %>%
    filter(
      !is.na(dosage_G),
      !is.na(outcome),
      is.finite(outcome),
      !is.na(mean_interval),
      !is.na(n_intervals)
    )
  if (nrow(dat) < 15 || length(unique(dat$dosage_G)) < 2) {
    return(data.frame(
      population = breed, gene = gene, trait = trait,
      n_ewes = nrow(dat),
      estimate = NA_real_, se = NA_real_, statistic = NA_real_,
      df = NA_real_, p_value = NA_real_,
      conf_low = NA_real_, conf_high = NA_real_
    ))
  }
  fit <- lm(
    log1p(outcome) ~ dosage_G + mean_interval + n_intervals,
    data = dat
  )
  extract_lm_term(fit, "dosage_G") %>%
    mutate(
      population = breed,
      gene = gene,
      trait = trait,
      n_ewes = nrow(dat),
      .before = 1
    )
}
stability_additive_results <- purrr::cross_df(list(
  population = c("Hu", "Tan"),
  gene = genes,
  trait = stability_traits
)) %>%
```

```
  mutate(
    result = pmap(
      list(population, gene, trait),
      ~ fit_stability_additive(ewe_stability, ..1, ..2, ..3)
    )
  ) %>%
  select(result) %>%
  unnest(result) %>%
  mutate(
    p_FDR = p.adjust(p_value, method = "BH"),
    effect_scale = "Change in log1p(stability trait) per G allele"
  )
hu_cd226_stability <- ewe_stability %>%
  filter(population == "Hu", !is.na(CD226)) %>%
  mutate(
    CD226_group = if_else(CD226 == "AA", "AA", "AG+GG"),
    CD226_group = factor(CD226_group, levels = c("AA", "AG+GG"))
  )
fit_hu_cd226_stability <- function(trait) {
  dat <- hu_cd226_stability %>%
    mutate(outcome = .data[[trait]]) %>%
    filter(!is.na(outcome), is.finite(outcome))
  fit <- lm(
    log1p(outcome) ~ CD226_group + mean_interval + n_intervals,
    data = dat
  )
  extract_lm_term(fit, "CD226_groupAG+GG") %>%
    mutate(
      population = "Hu",
      gene = "CD226",
      trait = trait,
      comparison = "AG+GG vs AA",
      n_ewes = nrow(dat),
      .before = 1
    )
}
hu_cd226_stability_results <- map_dfr(
  stability_traits,
  fit_hu_cd226_stability
) %>%
  mutate(p_FDR = p.adjust(p_value, method = "BH"))
RUN_PERMUTATION <- TRUE
N_PERM <- 10000
```

```
permutation_test_cd226 <- function(trait, n_perm = 10000) {
  dat <- hu_cd226_stability %>%
    mutate(outcome = .data[[trait]]) %>%
    filter(!is.na(outcome), is.finite(outcome))
  obs_fit <- lm(
    log1p(outcome) ~ CD226_group + mean_interval + n_intervals,
    data = dat
  )
  obs_beta <- coef(obs_fit)[["CD226_groupAG+GG"]]
  perm_beta <- replicate(n_perm, {
    dat_perm <- dat
    dat_perm$CD226_group <- sample(dat_perm$CD226_group)
    fit_perm <- lm(
      log1p(outcome) ~ CD226_group + mean_interval + n_intervals,
      data = dat_perm
    )
    coef(fit_perm)[["CD226_groupAG+GG"]]
  })
  data.frame(
    trait = trait,
    observed_beta = obs_beta,
    n_perm = n_perm,
    permutation_p = (sum(abs(perm_beta) >= abs(obs_beta), na.rm = TRUE) + 1) /
      (sum(!is.na(perm_beta)) + 1)
  )
}
if (RUN_PERMUTATION) {
  hu_cd226_permutation <- map_dfr(
    stability_traits,
    ~ permutation_test_cd226(.x, N_PERM)
  )
} else {
  hu_cd226_permutation <- data.frame()
}
genotype_long <- gt_all %>%
  pivot_longer(
    cols = all_of(genes),
    names_to = "gene",
    values_to = "genotype"
  ) %>%
  filter(!is.na(genotype)) %>%
  mutate(
    genotype = factor(genotype, levels = c("AA", "AG", "GG"))
  )
```

```
genotype_frequency <- genotype_long %>%
  count(population, gene, genotype, name = "n") %>%
  group_by(population, gene) %>%
  mutate(
    total = sum(n),
    frequency = n / total
  ) %>%
  ungroup()
calc_genetic_stats <- function(df, population_name, gene_name) {
  total_genotyped <- gt_all %>%
    filter(population == population_name) %>%
    nrow()
  g <- df %>%
    filter(population == population_name, gene == gene_name) %>%
    pull(genotype) %>%
    as.character()
  g <- g[!is.na(g)]
  nAA <- sum(g == "AA")
  nAG <- sum(g == "AG")
  nGG <- sum(g == "GG")
  N <- length(g)
  if (N == 0) {
    return(data.frame(
      population = population_name, gene = gene_name,
      N = 0, call_rate = 0,
      nAA = 0, nAG = 0, nGG = 0,
      freq_A = NA_real_, freq_G = NA_real_,
      Ho = NA_real_, He = NA_real_,
      Ne = NA_real_, PIC = NA_real_, HWE_P_exact = NA_real_
    ))
  }
  freq_A <- (2 * nAA + nAG) / (2 * N)
  freq_G <- (2 * nGG + nAG) / (2 * N)
  Ho <- nAG / N
  He <- 1 - (freq_A^2 + freq_G^2)
  Ne <- 1 / (freq_A^2 + freq_G^2)
  PIC <- 1 - (freq_A^2 + freq_G^2) - 2 * (freq_A^2 * freq_G^2)

  hwe_p <- tryCatch(
    HardyWeinberg::HWExact(c(nAA, nAG, nGG), verbose = FALSE)$pval,
    error = function(e) NA_real_
  )
  data.frame(
    population = population_name,
```

```
        gene = gene_name,
        N = N,
        call_rate = N / total_genotyped,
        nAA = nAA,
        nAG = nAG,
        nGG = nGG,
        freq_A = freq_A,
        freq_G = freq_G,
        Ho = Ho,
        He = He,
        Ne = Ne,
        PIC = PIC,
        HWE_P_exact = hwe_p
    )
}
genetic_stats <- purrr::cross_df(list(
    population = c("Hu", "Tan"),
    gene = genes
)) %>%
    mutate(
        result = map2(
            population, gene,
            ~ calc_genetic_stats(genotype_long, .x, .y)
        )
    ) %>%
    select(result) %>%
    unnest(result)
allele_frequency <- genetic_stats %>%
    select(population, gene, freq_A, freq_G) %>%
    pivot_longer(
        cols = c(freq_A, freq_G),
        names_to = "allele",
        values_to = "frequency"
    ) %>%
    mutate(
        allele = dplyr::recode(allele, "freq_A" = "A", "freq_G" = "G")
    )
population_cols <- c(
    "Hu"    = "#0072B2",
    "Tan" = "#D55E00"
)
genotype_cols <- c(
    "AA" = "#0072B2",
    "AG" = "#B8B8B8",
```

```
  "GG" = "#D55E00"
)
allele_cols <- c(
  "A" = "#0072B2",
  "G" = "#D55E00"
)
theme_pub_clean <- function(base_size = 11) {
  theme_classic(base_size = base_size, base_family = "sans") +
    theme(
      axis.title = element_text(size = base_size + 1, face = "bold"),
      axis.text = element_text(size = base_size, colour = "black"),
      strip.background = element_rect(
        fill = "#F2F2F2",
        colour = "black",
        linewidth = 0.45
      ),
      strip.text = element_text(
        size = base_size + 0.5,
        face = "bold",
        colour = "black"
      ),
      legend.title = element_blank(),
      legend.text = element_text(size = base_size),
      legend.key = element_blank(),
      plot.title = element_blank(),
      plot.subtitle = element_blank(),
      plot.caption = element_blank(),
      plot.tag = element_text(size = base_size + 3, face = "bold"),
      panel.border = element_rect(
        fill = NA,
        colour = "black",
        linewidth = 0.45
      ),
      axis.line = element_blank(),
      plot.margin = margin(6, 8, 6, 6)
    )
}
theme_set(theme_pub_clean())
save_pub <- function(plot_object, file_stub, width = 8.5, height = 6.5) {

  tiff_file <- file.path(fig_dir, paste0(file_stub, ".tiff"))
  png_file    <- file.path(fig_dir, paste0(file_stub, ".png"))
  pdf_file    <- file.path(fig_dir, paste0(file_stub, ".pdf"))
  ragg::agg_tiff(
```

```r
        filename = tiff_file,
        width = width,
        height = height,
        units = "in",
        res = 1000,
        compression = "lzw",
        background = "white"
    )
    print(plot_object)
    dev.off()
    ragg::agg_png(
        filename = png_file,
        width = width,
        height = height,
        units = "in",
        res = 1000,
        background = "white"
    )
    print(plot_object)
    dev.off()
    ggsave(
        filename = pdf_file,
        plot = plot_object,
        width = width,
        height = height,
        units = "in",
        device = grDevices::cairo_pdf,
        bg = "white"
    )
}
ewes_with_2plus <- lambing_clean %>%
    filter(!is.na(lambing_date)) %>%
    count(population, ewe_id, name = "n_lambings") %>%
    filter(n_lambings >= 2) %>%
    count(population, name = "ewes_with_2plus")
raw_adjacent_intervals <- interval_all %>%
    filter(adjacent_parity) %>%
    count(population, name = "raw_adjacent_intervals")
valid_summary_plot <- valid_intervals %>%
    summarise(
        valid_intervals = n(),
        valid_ewes = n_distinct(ewe_id),
        .by = population
    )
```

```
qc_flow_plot <- gt_all %>%
  count(population, name = "genotyped_ewes") %>%
  left_join(ewes_with_2plus, by = "population") %>%
  left_join(raw_adjacent_intervals, by = "population") %>%
  left_join(valid_summary_plot, by = "population")
flow_long <- qc_flow_plot %>%
  pivot_longer(
    cols = c(
      genotyped_ewes,
      ewes_with_2plus,
      raw_adjacent_intervals,
      valid_intervals,
      valid_ewes
    ),
    names_to = "stage",
    values_to = "n"
  ) %>%
  mutate(
    stage = factor(
      stage,
      levels = c(
        "genotyped_ewes",
        "ewes_with_2plus",
        "raw_adjacent_intervals",
        "valid_intervals",
        "valid_ewes"
      ),
      labels = c(
        "Genotyped\newes",
        "Ewes with ≥2\nvalid dates",
        "Adjacent-parity\nintervals",
        "Valid intervals\n(180–450 d)",
        "Ewes contributing\nvalid intervals"
      )
    ),
    population = factor(population, levels = c("Hu", "Tan"))
  )
p1a <- ggplot(
  flow_long,
  aes(x = stage, y = population, group = population, colour = population)
) +
  geom_line(linewidth = 0.65, alpha = 0.70) +
  geom_label(
    aes(label = n, fill = population),
```

```r
        colour = "white",
        fontface = "bold",
        label.size = 0,
        size = 3.3
    ) +
    scale_colour_manual(values = population_cols) +
    scale_fill_manual(values = population_cols) +
    labs(x = NULL, y = NULL) +
    guides(colour = "none", fill = "none") +
    theme(
        panel.border = element_rect(fill = NA, colour = "black", linewidth = 0.45),
        axis.text.x = element_text(size = 9.5, lineheight = 0.95),
        axis.text.y = element_text(size = 10.5),
        axis.ticks = element_blank()
    )
p1b <- ggplot(
    valid_intervals,
    aes(x = interval_days, fill = population)
) +
    geom_histogram(
        binwidth = 10,
        boundary = 180,
        colour = "white",
        linewidth = 0.15
    ) +
    geom_vline(
        data = overall_summary,
        aes(xintercept = mean_days, colour = population),
        linetype = 2,
        linewidth = 0.75
    ) +
    facet_wrap(~ population, nrow = 1, scales = "free_y") +
    scale_fill_manual(values = population_cols) +
    scale_colour_manual(values = population_cols) +
    scale_x_continuous(
        limits = c(180, 450),
        breaks = seq(180, 450, by = 45)
    ) +
    labs(
        x = "Lambing interval (days)",
        y = "Number of interval records"
    ) +
    guides(fill = "none", colour = "none")
fig1_clean <- p1a / p1b +
```

```
  plot_layout(heights = c(0.85, 1.35)) +
  plot_annotation(tag_levels = "A")

save_pub(
  fig1_clean,
  "Figure1_QC_and_interval_distribution",
  width = 9.0,
  height = 7.6
)
monthly_lambing <- lambing_clean %>%
  filter(!is.na(lambing_date)) %>%
  mutate(month = lubridate::month(lambing_date)) %>%
  count(population, month, name = "n") %>%
  complete(
    population,
    month = 1:12,
    fill = list(n = 0)
  ) %>%
  mutate(population = factor(population, levels = c("Hu", "Tan")))

p2a <- ggplot(
  monthly_lambing,
  aes(x = month, y = n, colour = population, group = population)
) +
  geom_line(linewidth = 0.85) +
  geom_point(size = 2.0) +
  scale_colour_manual(values = population_cols) +
  scale_x_continuous(
    breaks = 1:12,
    labels = month.abb
  ) +
  labs(
    x = "Calendar month",
    y = "Number of lambing records"
  ) +
  theme(legend.position = "top")

p2b <- ggplot(
  valid_intervals,
  aes(x = prev_season, y = interval_days, fill = population)
) +
  geom_boxplot(
    position = position_dodge(width = 0.72),
    width = 0.58,
```

```
        outlier.shape = NA,
        alpha = 0.82,
        linewidth = 0.45
    ) +
    geom_point(
        aes(colour = population),
        position = position_jitterdodge(
            jitter.width = 0.10,
            dodge.width = 0.72
        ),
        size = 0.55,
        alpha = 0.20
    ) +
    scale_fill_manual(values = population_cols) +
    scale_colour_manual(values = population_cols) +
    labs(
        x = "Season of the preceding lambing",
        y = "Lambing interval (days)"
    ) +
    guides(fill = "none", colour = "none")
population_emmeans_plot <- population_emmeans %>%
    mutate(
        analysis_short = case_when(
            grepl("^Main", analysis) ~ "Main analysis",
            grepl("^Winter", analysis) ~ "Winter-matched",
            TRUE ~ analysis
        ),
        analysis_short = factor(
            analysis_short,
            levels = c("Main analysis", "Winter-matched")
        ),
        population = factor(population, levels = c("Hu", "Tan"))
    )
pd <- position_dodge(width = 0.35)

p2c <- ggplot(
    population_emmeans_plot,
    aes(
        x = analysis_short,
        y = emmean,
        colour = population,
        group = population
    )
) +
```

```r
  geom_errorbar(
    aes(ymin = lower.CL, ymax = upper.CL),
    position = pd,
    width = 0.10,
    linewidth = 0.65
  ) +
  geom_point(
    position = pd,
    size = 2.6
  ) +
  scale_colour_manual(values = population_cols) +
  labs(
    x = NULL,
    y = "Adjusted mean lambing interval (days)"
  ) +
  theme(
    legend.position = "top",
    axis.text.x = element_text(size = 9.5)
  )
fig2_clean <- p2a / (p2b | p2c) +
  plot_layout(heights = c(1.0, 1.12), widths = c(1.25, 1.0)) +
  plot_annotation(tag_levels = "A")
save_pub(
  fig2_clean,
  "Figure2_Seasonality_and_adjusted_population_effect",
  width = 10.0,
  height = 7.5
)
parity_n_plot <- parity_summary %>%
  mutate(
    population = factor(population, levels = c("Hu", "Tan")),
    label_y = if_else(population == "Hu", 274, 388),
    n_label = paste0("n=", n_intervals)
  )

p3 <- ggplot(
  parity_emmeans,
  aes(
    x = parity_group,
    y = emmean,
    colour = population,
    group = population
  )
) +
```

```r
  geom_errorbar(
    aes(ymin = lower.CL, ymax = upper.CL),
    width = 0.10,
    linewidth = 0.70
  ) +
  geom_line(linewidth = 0.80) +
  geom_point(size = 2.6) +
  geom_text(
    data = parity_n_plot,
    aes(
      x = parity_group,
      y = label_y,
      label = n_label,
      colour = population
    ),
    inherit.aes = FALSE,
    size = 3.0,
    fontface = "bold",
    show.legend = FALSE
  ) +
  scale_colour_manual(values = population_cols) +
  coord_cartesian(ylim = c(270, 390)) +
  labs(
    x = "Parity interval",
    y = "Adjusted lambing interval (days)"
  ) +
  theme(legend.position = "top")

save_pub(
  p3,
  "Figure3_Adjusted_parity_patterns",
  width = 8.5,
  height = 5.8
)
genotype_frequency_plot <- genotype_frequency %>%
  mutate(
    genotype = factor(genotype, levels = c("AA", "AG", "GG")),
    population = factor(population, levels = c("Hu", "Tan"))
  )
p4a <- ggplot(
  genotype_frequency_plot,
  aes(x = gene, y = frequency, fill = genotype)
) +
  geom_col(
```

```
    position = position_stack(reverse = TRUE),
    width = 0.70,
    colour = "white",
    linewidth = 0.25
  ) +
  facet_wrap(~ population, nrow = 1) +
  scale_fill_manual(values = genotype_cols, drop = FALSE) +
  scale_y_continuous(
    labels = percent_format(accuracy = 1),
    limits = c(0, 1),
    expand = expansion(mult = c(0, 0.015))
  ) +
  labs(
    x = NULL,
    y = "Genotype frequency"
  ) +
  theme(legend.position = "top")
allele_frequency_plot <- allele_frequency %>%
  mutate(
    allele = factor(allele, levels = c("A", "G")),
    population = factor(population, levels = c("Hu", "Tan"))
  )
p4b <- ggplot(
  allele_frequency_plot,
  aes(x = gene, y = frequency, fill = allele)
) +
  geom_col(
    position = position_dodge(width = 0.72),
    width = 0.62,
    colour = "black",
    linewidth = 0.25
  ) +
  facet_wrap(~ population, nrow = 1) +
  scale_fill_manual(values = allele_cols) +
  scale_y_continuous(
    labels = percent_format(accuracy = 1),
    limits = c(0, 1),
    expand = expansion(mult = c(0, 0.015))
  ) +
  labs(
    x = NULL,
    y = "Allele frequency"
  ) +
  theme(legend.position = "top")
```

```
fig4_clean <- p4a / p4b +
  plot_annotation(tag_levels = "A")

save_pub(
  fig4_clean,
  "Figure5B_C_Genotype_and_allele_frequencies",
  width = 9.0,
  height = 7.5
)
forest_data <- snp_additive_results %>%
  mutate(
    gene = factor(
      gene,
      levels = rev(c("ADAMTSL1", "CD226", "PAPPA"))
    ),
    population = factor(population, levels = c("Hu", "Tan"))
  )
x_lim <- ceiling(
  max(
    abs(c(forest_data$conf_low, forest_data$conf_high)),
    na.rm = TRUE
  )
)
p5 <- ggplot(
  forest_data,
  aes(
    x = estimate,
    y = gene,
    colour = population
  )
) +
  geom_vline(
    xintercept = 0,
    linetype = 2,
    colour = "#777777",
    linewidth = 0.55
  ) +
  geom_errorbar(
    aes(xmin = conf_low, xmax = conf_high),
    orientation = "y",
    width = 0.14,
    linewidth = 0.75
  ) +
  geom_point(size = 2.7) +
```

```r
  facet_wrap(~ population, nrow = 1) +
  scale_colour_manual(values = population_cols) +
  scale_x_continuous(
    limits = c(-x_lim, x_lim),
    breaks = pretty(c(-x_lim, x_lim), n = 5)
  ) +
  labs(
    x = "Adjusted change in lambing interval per G allele (days)",
    y = NULL
  ) +
  guides(colour = "none")

save_pub(
  p5,
  "Figure6_SNP_additive_effects_forest",
  width = 9.0,
  height = 5.2
)
cat("R-generated manuscript figures were written to: ", fig_dir, "\n")
stability_plot_data <- hu_cd226_stability %>%
  select(
    ewe_id, CD226_group,
    sd_interval, mad_interval, cv_interval
  ) %>%
  pivot_longer(
    cols = c(sd_interval, mad_interval, cv_interval),
    names_to = "trait",
    values_to = "value"
  ) %>%
  mutate(
    trait = factor(
      trait,
      levels = c("sd_interval", "mad_interval", "cv_interval"),
      labels = c("Interval SD (days)", "Interval MAD (days)", "Interval CV")
    )
  )
p_stability <- ggplot(
  stability_plot_data,
  aes(x = CD226_group, y = value, fill = CD226_group)
) +
  geom_boxplot(width = 0.62, outlier.shape = NA, alpha = 0.8) +
  geom_jitter(width = 0.10, size = 1.0, alpha = 0.45) +
  facet_wrap(~ trait, scales = "free_y", nrow = 1) +
  scale_fill_manual(values = c("AA" = "#4C78A8", "AG+GG" = "#E45756")) +
```

```
  labs(
    x = "CD226 genotype group",
    y = NULL,
    fill = "Genotype group",
    title = "Exploratory association between CD226 and lambing-interval stability in Hu sheep",
    subtitle = "Only ewes with at least three valid lambing intervals were included"
  ) +
  guides(fill = "none")

save_pub(p_stability, "Diagnostic_Hu_CD226_interval_stability", 10.0, 4.8)
write.csv(
  lambing_clean,
  file.path(data_dir, "clean_lambing_records.csv"),
  row.names = FALSE, fileEncoding = "UTF-8"
)
write.csv(
  interval_all,
  file.path(data_dir, "all_recalculated_intervals_with_QC.csv"),
  row.names = FALSE, fileEncoding = "UTF-8"
)
write.csv(
  valid_intervals,
  file.path(data_dir, "valid_intervals_180_450.csv"),
  row.names = FALSE, fileEncoding = "UTF-8"
)
write.csv(
  ewe_stability,
  file.path(data_dir, "ewe_level_stability_min3_intervals.csv"),
  row.names = FALSE, fileEncoding = "UTF-8"
)
write.csv(
  date_anomalies,
  file.path(data_dir, "date_and_litter_size_anomalies.csv"),
  row.names = FALSE, fileEncoding = "UTF-8"
)
write.csv(
  duplicate_parity_records,
  file.path(data_dir, "duplicate_ewe_parity_records.csv"),
  row.names = FALSE, fileEncoding = "UTF-8"
)
result_sheets <- list(
  QC_flow = qc_flow,
  Invalid_reasons = invalid_reason_summary,
  Date_anomalies = date_anomalies,
```

```r
    Overall_summary = overall_summary,
    Parity_summary = parity_summary,
    Population_effects = population_effects,
    Population_EMMs = population_emmeans,
    Parity_EMMs = parity_emmeans,
    Parity_model_ANOVA = parity_model_anova,
    SNP_additive = snp_additive_results,
    SNP_dominant_sensitivity = dominant_results,
    SNP_genotype_counts = snp_genotype_counts,
    Stability_sample = stability_sample_summary,
    Stability_additive = stability_additive_results,
    Hu_CD226_stability = hu_cd226_stability_results,
    Hu_CD226_permutation = hu_cd226_permutation,
    Genotype_frequency = genotype_frequency,
    Allele_frequency = allele_frequency,
    Genetic_stats_HWE = genetic_stats
)

writexl::write_xlsx(
    result_sheets,
    path = file.path(table_dir, "Statistical_analysis_results.xlsx")
)
model_summary_file <- file.path(model_dir, "model_summaries.txt")
sink(model_summary_file)
cat("============================================================\n")
cat("MAIN POPULATION MIXED MODEL\n")
cat("============================================================\n")
if (!is.null(main_model)) print(summary(main_model))
cat("\n============================================================\n")
cat("WINTER + COMMON YEARS SENSITIVITY MODEL\n")
cat("Common years: ", paste(common_years, collapse = ", "), "\n")
cat("============================================================\n")
if (!is.null(sensitivity_model)) print(summary(sensitivity_model))
cat("\n============================================================\n")
cat("POPULATION × PARITY MIXED MODEL\n")
cat("============================================================\n")
if (!is.null(parity_model)) {
    print(summary(parity_model))
    print(anova(parity_model))
}
cat("\n============================================================\n")
cat("SNP ADDITIVE RESULTS\n")
cat("============================================================\n")
print(snp_additive_results)
```

```
cat("\n============================================================\n")
cat("STABILITY RESULTS\n")
cat("============================================================\n")
print(stability_additive_results)
print(hu_cd226_stability_results)
print(hu_cd226_permutation)
cat("\n============================================================\n")
cat("SESSION INFORMATION\n")
cat("============================================================\n")
print(sessionInfo())
sink()
saveRDS(main_model, file.path(model_dir, "main_population_model.rds"))
saveRDS(sensitivity_model, file.path(model_dir, "winter_common_year_model.rds"))
saveRDS(parity_model, file.path(model_dir, "population_parity_model.rds"))
cat("\nAnalysis completed.\n")
cat("Output directory: ", out_dir, "\n\n")
cat("Quality-control workflow:\n")
print(qc_flow)
cat("\nOverall summary after applying the unified 180 – 450 d range:\n")
print(overall_summary)
cat("\nAdjusted population effects:\n")
print(population_effects)
cat("\nAdditive-model results for the three SNPs:\n")
print(snp_additive_results)
cat("\nSample size for the stability analysis:\n")
print(stability_sample_summary)
cat("\nNote: Figure 4 (PCR/Sanger images) and Figure 5A (PARMS cluster plots) are laboratory/instrument images and are not generated from the phenotype/genotype summary workbooks.\n")
```
